# *Albatrellus confluens* (Alb. & Schwein.) Kotl. & Pouz.: Natural Fungal Compounds and Synthetic Derivatives with In Vitro Anthelmintic Activities and Antiproliferative Effects against Two Human Cancer Cell Lines

**DOI:** 10.3390/molecules27092950

**Published:** 2022-05-05

**Authors:** Mthandazo Dube, Dayma Llanes, Mohamad Saoud, Robert Rennert, Peter Imming, Cécile Häberli, Jennifer Keiser, Norbert Arnold

**Affiliations:** 1 Department of Bioorganic Chemistry, Leibniz Institute of Plant Biochemistry, Weinberg 3, D-06120 Halle (Saale), Germany; mthandazo.dube@ipb-halle.de (M.D.); dayma.llanesmartinez@ipb-halle.de (D.L.); mohamad.saoud@ipb-halle.de (M.S.); robert.rennert@ipb-halle.de (R.R.); 2 Institute of Pharmacy, Faculty of Natural Sciences, Martin-Luther-University Halle-Wittenberg, D-06120 Halle (Saale), Germany; peter.imming@pharmazie.uni-halle.de; 3 Swiss Tropical and Public Health Institute, Kreuzstrasse 2, CH-4123 Allschwil, Switzerland; cecile.haeberli@swisstph.ch (C.H.); jennifer.keiser@swisstph.ch (J.K.); 4 University of Basel, CH-4051 Basel, Switzerland

**Keywords:** *Albatrellus confluens* (Alb. & Schwein.) Kotl. & Pouz., anthelmintic properties, schistosomiasis, anticancer activities

## Abstract

Neglected tropical diseases affect the world’s poorest populations with soil-transmitted helminthiasis and schistosomiasis being among the most prevalent ones. Mass drug administration is currently the most important control measure, but the use of the few available drugs is giving rise to increased resistance of the parasites to the drugs. Different approaches are needed to come up with new therapeutic agents against these helminths. Fungi are a source of secondary metabolites, but most fungi remain largely uninvestigated as anthelmintics. In this report, the anthelmintic activity of *Albatrellus confluens* against *Caenorhabditis elegans* was investigated using bio-assay guided isolation. Grifolin (**1**) and neogrifolin (**2**) were identified as responsible for the anthelmintic activity. Derivatives **4**–**6** were synthesized to investigate the effect of varying the prenyl chain length on anthelmintic activity. The isolated compounds **1** and **2** and synthetic derivatives **4**–**6**, as well as their educts 7–10, were tested against *Schistosoma mansoni* (adult and newly transformed schistosomula), *Strongyloides ratti*, *Heligmosomoides polygyrus*, *Necator americanus,* and *Ancylostoma ceylanicum.* Prenyl-2-orcinol (**4**) and geranylgeranyl-2-orcinol (**6**) showed promising activity against newly transformed schistosomula. The compounds **1**, **2**, **4**, **5,** and **6** were also screened for antiproliferative or cytotoxic activity against two human cancer lines, viz. prostate adenocarcinoma cells (PC-3) and colorectal adenocarcinoma cells (HT-29). Compound **6** was determined to be the most effective against both cell lines with IC_50_ values of 16.1 µM in PC-3 prostate cells and 33.7 µM in HT-29 colorectal cells.

## 1. Introduction

Neglected tropical diseases (NTDs) affect more than 1.7 billion of the world’s poorest populations. The two most common are the soil-transmitted helminthiases and schistosomiasis. Soil-transmitted helminths (STHs) are parasitic worm infections affecting mainly marginalized population groups in the tropics and subtropics. The STHs with the highest infection rates are the “large roundworm” *Ascaris lumbricoides*, the whipworm *Trichuris trichiura*, and the hookworms *Ancylostoma duodenale* (Old World hookworm) and *Necator americanus* (New World hookworm) which affect more than 1.5 billion people worldwide [1]. Schistosomiasis (snail fever, bilharzia) is caused by parasitic flatworms of the genus *Schistosoma* with about 240 million infected people worldwide and more than 700 million people living in endemic areas [2]. The coronavirus disease 2019 (COVID-19 pandemic) has not only caused the death of millions but has also severely disrupted health systems and economies throughout the world [3]. This resulted in the suspension of many health services and programs, including those for neglected tropical diseases (NTDs) [4]. The prevalence of NTDs including STHs probably increased due to the suspension of Mass Drug Administration (MDA) programs which were the main control measure for STHs [5] and schistosomiasis was identified as one of the diseases that was most affected by disruption of control programs [4]. Approaches tapping into neglected resources might therefore even be more required for the control of STHs and schistosomiasis. Moreover, the current control measure, which is MDA, makes synthetic anthelmintic drugs potential environmental contaminants [6]. The chemical residues from anthelmintic drugs affect dung beetles, bacteria, fungi, mites, and worms that live in the soil, through pollution of soil and water [7]. Lastly, MDA programs are also responsible for creating selective pressure on human parasites which will give rise to increasing levels of resistance [8]. The extensive use of anthelmintic drugs in the control of animal parasites leads to widespread anthelmintic resistance. The resistance to anthelmintic drugs in veterinary species serves as an indicator of how anthelmintic resistance of human parasites may increase [9]. Although in humans there is no standardized surveillance protocol for monitoring resistance to anthelmintic drugs in places where mass drug administration takes place [10], there is already evidence of anthelmintic drug resistance. In Uganda, the efficacy of praziquantel has been shown to be lower in schools that have a longer duration of mass drug administration [11]. Another example is in Pemba Island, where poor efficacy of albendazole against *T. trichiura* and hookworms has been reported. Pemba Island has a long history of MDA [12]. Drug resistance is an emerging problem and there is, therefore, a need for new natural product-based anthelmintics to help reduce the global prevalence of human nematode infections. 

One approach could be to look at fungi as a possible source of anthelmintic compounds. There are 2.2–3.8 million species of fungi worldwide [13] and of these, between 140,000–160,000 are mushrooms and only about 10% of the mushrooms have been investigated [14,15]. Mushrooms have been used across the globe for their medicinal properties and they are sources of potent pharmaceuticals [16]. Edible mushrooms have shown anthelmintic activity against *Haemonchus contortus*, which is also known as the barber’s pole worm, and is a very common parasite and one of the most pathogenic nematodes of ruminants [17]. Recently, a cyclodepsipeptide PF1022A, isolated from fungal culture *Mycelia sterilia* showed anthelmintic activity against *Ascaridia galli* in chickens [18]. Emodepside, a semisynthetic derivative of PF1022A, has shown efficacy against a variety of gastrointestinal nematodes in animals including but not limited to *H. contortus* (sheep), *Heterakis spumosa* (mice), *Cooperia oncophora* (cattle), and *Ascaris suum* (pigs) [19]. Emodepside is currently under clinical development for the treatment of onchocerciasis [20]. A study has shown that ten species of gilled fungi including the oyster mushroom *Pleurotus ostreatus* attack and consume nematodes [21]. The ability of edible mushrooms to produce nematocidal compounds that immobilize nematodes has been confirmed [21,22]. A fraction from the hydro-ethanolic extract of *Pleurotus djamor*, which is also an edible mushroom, showed ovicidal activity against *H. contortus* [23]. The red pigment from *Aspergillus terreus* has also exhibited anthelmintic activity against *H. contortus* [24]. The reason for the various biological activities of fungi may be due to the fact that fungal fruiting bodies are a highly demanded food and habitat source in an ecosystem. A high number of symbiotic relationships between fungi and invertebrates or vertebrates are known to exist. For example, while feeding on fungal fruiting bodies or feeding on fungi, organisms ingest spores and ensure their dissemination over a greater distance [25]. In parallel, some fungi produce secondary metabolites which can protect the asco- and basidiomatas against different predators. These chemical constituents act either as constitutive defense compounds with toxic, bitter, or pungent properties or as wound-activated defense compounds which convert inactive precursors enzymatically to the active agent [26]. Half of the fungal biomass in the soil is consumed by nematodes. Therefore, fungi possess defense compounds as a means to protect themselves [27]. Fungi are therefore a good source of anthelmintic compounds and in some respect, fungi are a better alternative to vascular plants as sources of naturally occurring antinematodal compounds due to the less complex anatomical structure of fungi and their ability to adapt to growth in large fermenters [28]. Compounds isolated from plants may present some cost and sustainability issues, for example, if the compound is found in a slow-growing plant or found in tissues that cannot be sustainably harvested like root bark [29].

In this report, the extract of the fruiting bodies of eleven fungal species was assayed for in vitro anthelmintic activity using *Caenorhabditis elegans.* The species were selected based on observations that their fruiting bodies were usually not attacked by insect larvae in the field which is why we assume that they may contain biologically active compounds. Fruiting bodies of one species, *Albatrellus confluens* (Alb. & Schwein.) Kotl. & Pouz. showed anthelmintic activity. Using bioassay-guided isolation, we isolated grifolin (**1**) and neogrifolin (**2**) which were responsible for the anthelmintic activity. As anthelmintic compounds do not generate high economic returns, a low-cost synthesis is crucial for commercial development [29]. Therefore, we synthesized analogs of grifolin (**1**), namely prenyl-2-orcinol (**4**), geranyl-2-orcinol (**5**), and geranylgeranyl-2-orcinol (**6**) in one step reactions. Synthesized compounds **4**–**6** together with the educts orcinol (**3**)**,** prenol (**7**), geraniol (**8**), farnesol (**9**), and geranylgeraniol (**10**) were evaluated against *C. elegans*, followed by assaying against the five human and animal parasites *Schistosoma mansoni* (adult and newly transformed schistosomula), *Strongyloides ratti*, *Heligmosomoides polygyrus*, *Necator americanus*, and *Ancylostoma ceylanicum* (all larval stages). 

## 2. Results

**Figure 1:**
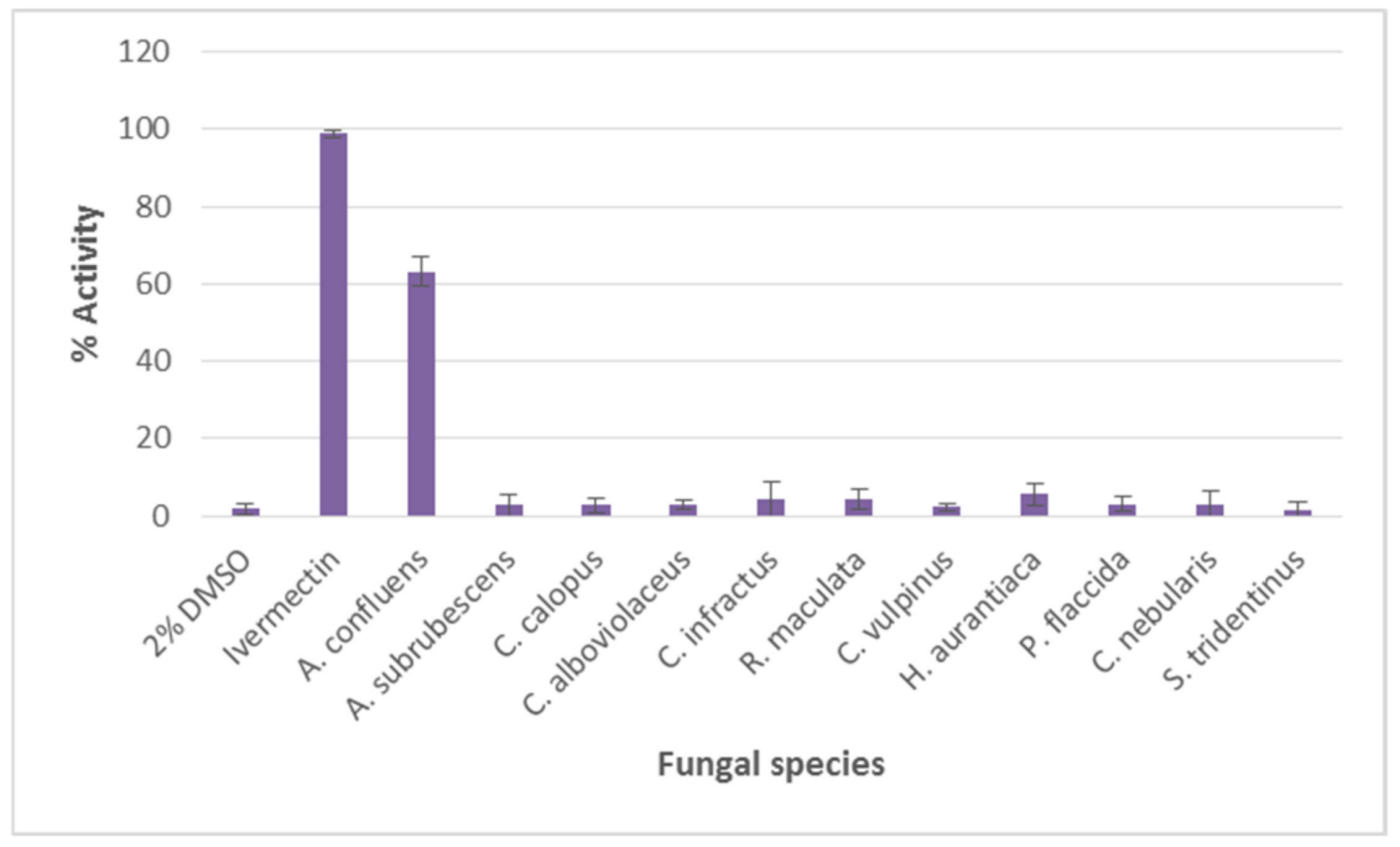
In vitro anthelmintic activity of crude extracts (500 µg/mL) of fungal fruiting bodies against *C. elegans*.

The crude extracts (80% aqueous methanol) from fruiting bodies of *Albatrellus confluens* (Alb. & Schwein.) Kotl. & Pouzar, *Albatrellus subrubescens* (Murr.) Pouz., *Caloboletus calopus* (Pers.) Vizzini, *Rhodocollybia maculata* (Alb. & Schwein.) Sing., *Cortinarius alboviolaceus* (Pers.) Fr., *Cortinarius infractus* (Pers.) Fr., *Cortinarius vulpinus* (Velen.) R. Hry., *Hygrophoropsis aurantiaca* (Wulfen) Maire, *Paralepista flaccida* (Sow.) Vizzini, *Clitocybe nebularis* (Batsch) P. Kumm. and *Suillus tridentinus* (Bres.) Sing. were tested for their anthelmintic properties against *C. elegans* as described before [30]. Only *A. confluens* showed anthelmintic activity above 50% while all other species tested had anthelmintic activity below 10% (Figure 1). The percentage of anthelmintic activity is the percentage of dead worms.

### A. confluens: Anthelmintic Activity of Crude Extract, Fractions and Metabolites

The mushroom showing activity, *A. confluens*, belongs to the family Albatrellaceae (Russulales) and is widely distributed across Europe, North America, and Asia. The mycorrhizal species grow in a variety of habitats but mainly occur in coniferous forests (*Pinus* spp., *Picea* spp.). The fruiting cap is pale peach-colored and circular with a diameter of 3–20 cm. The spores are cream-colored, the stipe up to 11 cm long and 3 cm thick [31].

**Figure 2:**
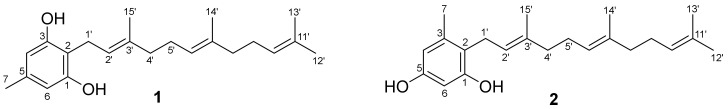
Structures of the naturally occurring compounds **1** and **2**.

The in vitro anthelmintic activity of 80% methanol crude extracts of fruiting bodies of *A. confluens* was evaluated using *C. elegans* as a model organism and revealed that the extract had good anthelmintic activity, killing 63.2 ± 3.8% of the nematodes at a concentration of 500 µg/mL. After partitioning the crude extract between water and different organic solvents, the anthelmintic activity of the organic fractions at a concentration of 500 µg/mL showed a killing rate against *C. elegans* of 72.3 ± 3.4% for *n*-hexane, 62.8 ± 4.5% for chloroform, and 75 ± 3.5% for ethyl acetate. The *n*-butanol and remaining water fraction had an activity of 17.1 ± 2.8% *(n*-butanol fraction) and 0.6 ± 0.9% (water fraction). The *n*-hexane, chloroform, and ethyl acetate fractions were combined and subjected to column chromatography on silica gel using a mixture of *n*-hexane:ethyl acetate (increasing polarity) as eluents. Using bioassay-guided fractionation, compound **1** (Figure 2) was isolated as an orange solid and identified as grifolin (**1**) (5-methyl-2-[(2*E*,6*E*)-3,7,11-trimethyldodeca-2,6,10-trienyl]benzene-1,3-diol) based on the spectral data, mainly ESI-HRMS, 1D, and 2D NMR (Figures S1–S6, Table S1; Supplementary Materials) and by comparison with detailed reported data [32,33,34]. Compound **2** (Figure 2) was isolated as a red oil and identified as neogrifolin (**2**) (5-methyl-4-[(2*E*,6*E*)-3,7,11-trimethyldodeca-2,6,10-trienyl]benzene-1,3-dio) by comparing its spectral data (Figures S7–S13, Table S2; Supplementary Materials) with those reported in the literature [32,33,34].

Compounds **1** and **2** were tested at a concentration of 500 µg/mL against *C. elegans* and compound **1** showed only moderate anthelmintic activity killing 32 ± 4.8% while compound **2** showed strong anthelmintic activity killing 100% of the worms and had an LC_50_ 410.6 µg/mL (Figure S34; Supplementary Materials).

**Scheme 1:**
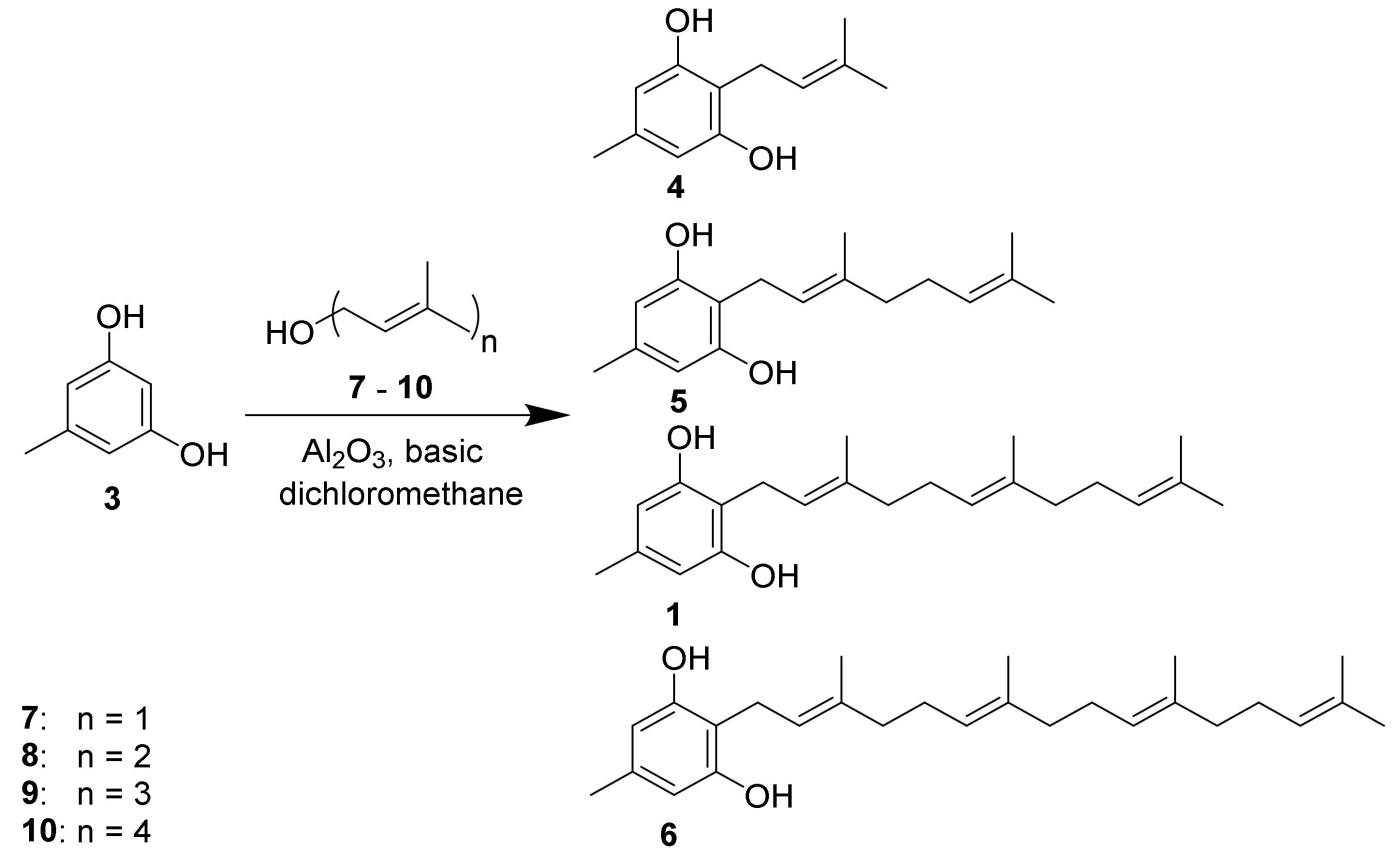
Synthesis of compounds **1**, **4**–**6** by regioselective alkylation of orcinol (**3**).

**Figure 3:**
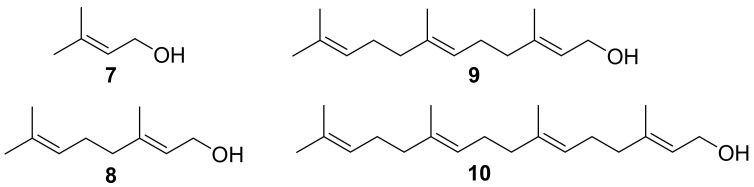
The prenyl alcohols **7**–**10** used in the synthesis of the analogs.

To assess the influence of the prenyl side chain in compounds **1** and **2** on their anthelmintic activities, derivatives of compound **1** with different side-chain lengths were synthesized in an alumina-promoted regioselective aromatic allylation one-step reaction adopted from Jentsch et al. [35] (Scheme 1; Figure 3).

The structures of the prenylated orcinol (**3**) derivatives, prenyl-2-orcinol (**4**) (5-methyl-2-(3-methylbut-2-enyl)benzene-1,3-diol), geranyl-2-orcinol (**5**) (2-[(2*E*)-3,7-dimethylocta-2,6-dienyl]-5-methylbenzene-1,3-diol), and geranylgeranyl-2-orcinol (**6**) (5-Methyl-2-(3,7,11,15-tetramethyl-2,6,10,14-hexadecatetraen-1-yl)-1,3-benzen) were confirmed by their ESI-HRMS, 1D and 2D NMR data (Supplementary Materials: **4**: Figures S14–S18, Table S3; **5** Figures S23–S25, Table S4; **6:** Figures S26–S33, Table S5). The educts of the synthesis of **1**, **3–6,** namely, orcinol (**3**) together with the prenyl alcohols prenol (**7**), geraniol (**8**), farnesol (**9**), and geranylgeraniol (**10**), were also tested against *C. elegans* at a concentration of 500 µg/mL. Orcinol (**3**) showed no activity against *C. elegans* while synthesized compounds geranyl-2-orcinol (**5**) and geranylgeranyl-2-orcinol (**6**) exhibited similar activity to their corresponding prenyl alcohols geraniol (**8**) and geranylgeraniol **10** (see Table 1 below). Only compound **4** had a much higher anthelmintic activity compared to prenol (**7**).

All compounds were tested in vitro against parasitic helminths. Compounds **1**, **2**, and **4**–**6** were tested against *S. mansoni* (adult and newly transformed schistosomules (NTS)), and the larval stages of *N. americanus*, *S. ratti*, *H. polygyrus*, and *Ancylostoma ceylanicum* at a concentration of 10 µM. Reference compounds were auranofin for NTS, praziquantel for adult *S. mansoni* and levamisole for *N. americanus*, *S. ratti*, *H. polygyrus* and abamectin for *A. ceylanicum* (all at 10 µM). Compounds showing anthelmintic activity above the threshold of 60% against the parasitic organisms were also tested at 1 µM (Table 2).

Compounds **4** and **6** showed good activity against newly transformed schistosomula, affecting 93.3% and 75.0% of the organisms at a concentration of 10 µM. At a concentration of 1 µM, compound **4** and compound **6** had a reduced activity rate of 38.9% and 55.0% against NTS. All compounds showed moderate, weak to no activity with respect to the other four parasitic organisms. Against adult *S. mansoni*, compounds **1**, **2**, and **4** had activities between 27 to 35% while compounds **5** and **6** had less than 20% activity. Compounds **5** and **6** had similar activity against *N. americanus* killing about 30% of the worms while compounds **1**, **2,** and **4** killed less than 20%. Compounds **5** and **6** showed moderate activity against *H. polygyrus* with almost 40% activity. Except for compound **4**, showing weak activity (24.4%), compounds **1**, **2**, **5,** and **6** were almost inactive against *A. ceylanicum.*

The prenyl alcohols **7**–**10** showed no significant activity against the parasites NTS, *N. americaus*, *S. ratti*, *H. polygyrus*, and *A. ceylanicum*. Reference compounds were auranofin for NTS and levamisole for *N. americanus*, *S. ratti*, *H. polygyrus* and abamectin *A. ceylanicum* (all at 10 µM) (Table 3). They were not tested against adult *S. mansoni* as the activity against NTS was less than 50%.

Compounds **1**, **2**, **4**, **5,** and **6** were screened for their potential antiproliferative and cytotoxic activity against two human cancer lines, namely prostate adenocarcinoma cells (PC-3) and colorectal adenocarcinoma cells (HT-29). The compounds’ effect on the metabolic cancer cell viability was determined by conducting a MTT assay, general cytotoxic effects were determined by using a CV assay, both after a 48-h cancer cell treatment with increasing concentrations of up to 100 µM of the compounds. The MTT and CV assay read-outs were normalized to 100% cell viability (0.5% DMSO negative control) and 0% cell viability (digitonin positive control) and analyzed by non-linear curve regression and IC_50_ values calculation using GraphPad Prism 8 software. 

**Figure 4:**
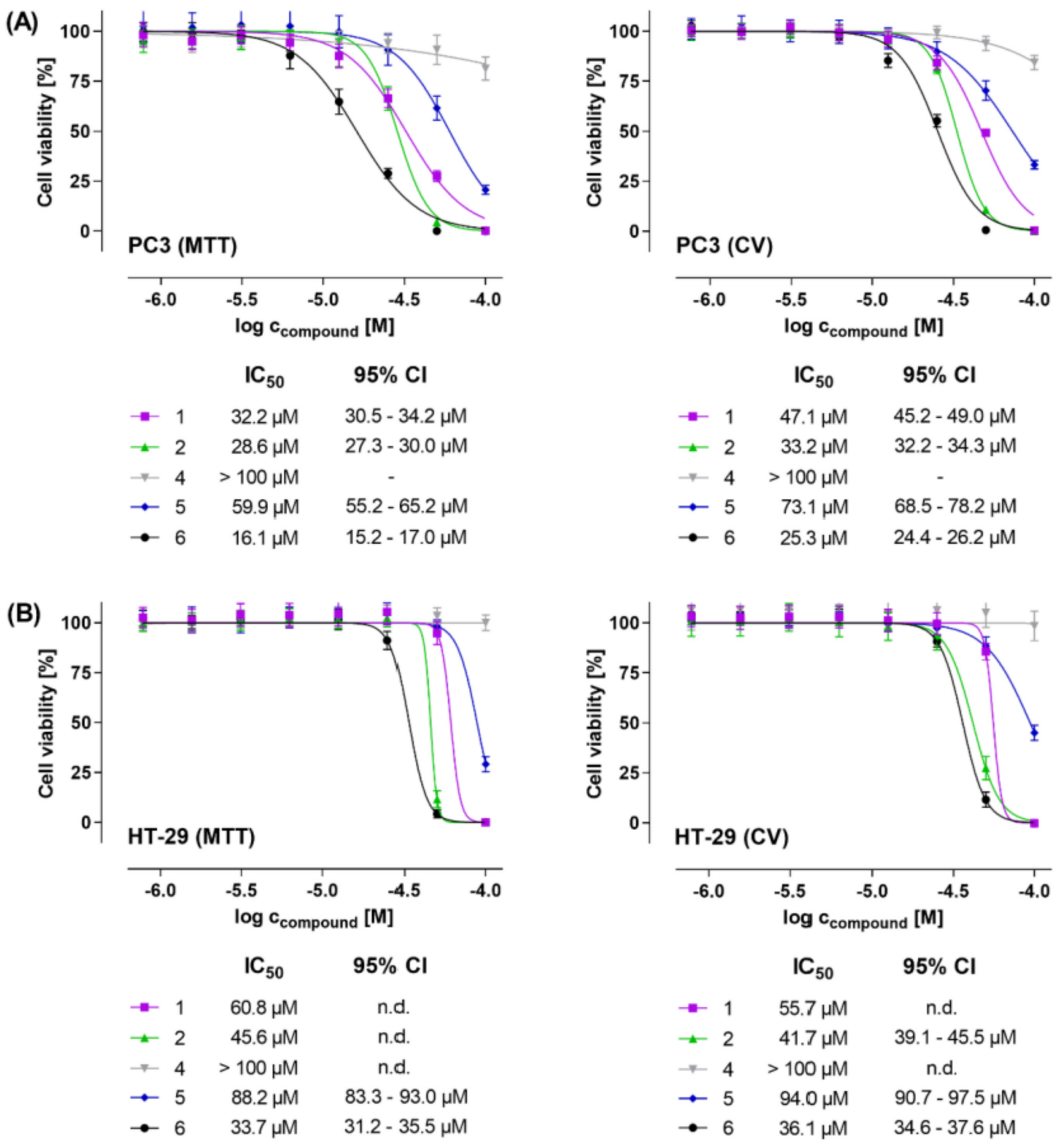
Effect of the compounds **1**, **2**, **4**, **5,** and **6** on the metabolic cell viability of (**A**) prostate PC-3 and (**B**) colorectal HT-29 cancer cells, respectively, as determined by MTT assay (**left**), and general cytotoxic and antiproliferative effect as determined by using crystal violet (CV) assay (**right**) after 48 h cell treatment. Data represent biological triplicates, each comprising technical quadruplicates. IC_50_ curves were analyzed and drawn using GraphPad Prism software. IC_50_ values are given with its calculated 95% confidence intervals (95% CI); n.d.: mathematically not definable based on the available data.

As shown in Figure 4, compound **4** lacked any substantial antiproliferative activity in both cell lines and both assays, as indicated by IC_50_ values > 100 µM. Whereas the compounds **1**, **2**, **5,** and **6** were detected to permit antiproliferative, and ultimately cytotoxic activity, in PC-3 and HT-29 cells with IC_50_ values in the range of 15–95 µM.

## 3. Discussion

Phytochemical investigations on fruiting bodies of *A. confluens* have been extensively reported [36,37,38,39] and also secondary metabolites of different compound classes are described from cultures of this mushroom [40,41,42]. In general, mushrooms of the genus *Albatrellus* including *A. confluens*, are well known for producing monomeric farnesylphenols, such as grifolin (**1**) and neogrifolin (**2**). The main constituent **1** of *A. confluens* was first described from the misnamed mushroom *Grifola confluens* [43]. Neogriofolin (**2**), which was first reported from the organic-chemical synthesis of grifolin (**1**) [44] was later recognized in different *Albatrellus* species [36,45]. 

The monomeric grifolin (**1**) and neogrifolin (**2**) are well known to possess diverse biological activities [45] such as anti-oxidative activity, anti-microbial effect, activity on human and rat vanilloid receptor 1, inhibition of tumor cell growth, promotion of melanin synthesis by B 16 melanin, inhibition of nitric oxide production in RAW 264.7 cells, the anti-cholesteremic activity level in blood and liver and plant growth inhibition. For compound **1**, a significant in vitro activity against three *Leishmania* species and *Trypanosoma cruzi* was reported [46].

The isomeric compounds grifolin (**1**) and neogrifolin (**2**) showed activity against *C. elegans* with neogrifolin (**2**) exhibiting higher activity against the free-living nematode. The higher activity may be due to the shift of the hydroxyl group at C-3 in grifolin (**1**) to C-5 in neogrifolin (**2**) and the shift of the methyl substituent in **1** from C-5 to C-3 in **2**. In general, phenolic compounds possess biological activity based on the ability to form a phenoxy radical which can then scavenge free radicals [47]. The anthelmintic activity observed against *C. elegans* was, however, probably not due to the antioxidant activity of the phenoxy anion as orcinol (**3**) showed no activity against *C. elegans*, while the activity seemed to be due to the presence of the prenyl chain as we demonstrated through the tested prenyl alcohols **7**–**10** (Table 2). From the activity profile of synthesized derivatives **4**–**6** of the farnesylphenod grifolin (**1**), it can be concluded that increasing prenyl chain length *n* (Scheme 1) reduced the anthelmintic activity against *C. elegans*. Compounds **4** (*n* = 1) and **5** (*n* = 2) bear shorter prenyl side chains than compound **1** (*n* = 3) and are more active while compound **6** (*n* = 4) exhibited almost no activity against *C. elegans*. There was also similar activity between the prenyl alcohols **8**–**10** and their corresponding phenolic compounds **1, 5**, and **6** except for compound **4** whereby the corresponding prenyl alcohol **7** had lower activity. Prenol (**7**) is known to elicit behavioral responses in *C. elegans* through the AWC neurons [48]. The observed anthelmintic activity may be due to the interaction of **4** with these neurons. The higher activity of **4** compared to the corresponding alcohol **7** (prenol) against *C. elegans* may also be explained by the fact that prenol (**7**) itself is a very small molecule, but as a substituent in **4** is part of a larger molecule that can bind more easily to the AWC receptor.

Previously, anthelmintic activity against *C. elegans* for geraniol (**8**), which is the main component in the essential oil of *Cymbopogon martini*, and other *Cymbopogon* species, has been reported [49,50]. The essential oils of *C. martini* and *Thymus bovei* have also shown anthelmintic activity against adult Indian earthworms *Pheretima posthuma* and this was attributed to the high content of geraniol (**8**) in the oils of both species [51,52]. Farnesol (**9**) has also been reported to have anthelmintic properties against *C. elegans* but is less potent than the monoterpenoid **8** [53] as also seen in our results. Furthermore, farnesol (**9**) exhibited nematicidal properties against the human parasitic *Anisakis* species which infects individuals who eat raw fish contaminated with the parasite [54]. Geranylgeraniol (**10**) isolated from *Pterodon pubescens* seed oil showed antitrypanosomal activity against bloodstream trypomastigotes of *Trypanosoma cruzi* [55]. In traditional medicine, terpenes play an important role as they are thought to be responsible for the anthelmintic properties of some plants such as Asian wormwood (*Artemisia annua*) and American wormseed (*Dysphania anthelmintica*) [56]. The use of terpenes as anthelmintics is however challenging as they are rapidly absorbed in the stomach and proximal intestine and therefore large doses are required to reach the target site where the intestinal parasites reside, resulting in toxic side effects [57]. The prenyl alcohols showed weak in vitro anthelmintic activity against the parasitic helminths (Table 3) and no correlation could be made between the in vitro anthelmintic activity of compounds **1**, **4**, **5**, and **6** and their corresponding prenyl alcohols.

The NTS against which compounds **4** and **6** showed good activity, is a target for drug and vaccine development as it is susceptible to the immune response [58]. Praziquantel kills adult worms and compounds that affect NTS and juvenile worms would offer a complementary approach in the fight against schistosomiasis. The activity shown by compounds **4** and **6** against NTS could therefore be a starting point for the development of similar compounds with modifications to enhance activity and bioavailability at the site of infection. As human schistosomiasis is only second to malaria in mortality [59] the importance of finding new alternative therapeutic agents to the current drug used cannot be overemphasized.

In addition to the in vitro anthelmintic screening, compounds **1**, **2**, **4**, **5**, and **6** were tested in vitro for their effects on the viability and proliferation of human cancer cell lines. Since two of the compounds, namely **1** (grifolin) and **2** (neogrifolin), have been tested and described by others, amongst others, for their impact on human HT-29 colorectal cancer cells [34], we decided to use the same cell line for our screenings, as well as human PC-3 prostate cancer cells as one of our most frequently used human cancer cell lines. Both allow comparison of the data with published data of others and with internal data of other natural products. 

Compound **6** was detected in vitro to be the most active of these compounds affecting cancer cells’ viability with IC_50_ values of 16.1 µM in PC-3 prostate cells and 33.7 µM in HT-29 colorectal cells (both in MTT assay), followed by the compounds **2**, **1** and **5**, in that order. In all cases, the respective IC_50_ value was lower by 1.5–2 in PC-3 prostate cancer cells compared to HT-29 colorectal cancer cells. However, the IC_50_ values determined in our study are in very good agreement with data very recently published, at least for compounds **1** and **2** [34]. Furthermore, the calculated IC_50_ values based on our MTT and CV data are very similar, with slightly lower IC_50_ values in the metabolic MTT cell viability assay. This indicates that metabolic cell viability is affected by compounds **1**, **2**, **5**, and **6**, triggering a cytotoxic effect that is ultimately leading to cancer cell death.

As mentioned above, cytotoxic activities toward human cancer cells of compounds **1** (grifolin) and **2** (neogrifolin) have been described by others. Yaqoob and coworkers, for instance, tested these compounds for anti-cell viability activity against human colon cancer cells, and published IC_50_ values in a quite narrow range of ~25–35 µM for all cell lines they tested [34], in very good accordance with the antiproliferative and cytotoxic activities, respectively, described here. The cancer cells’ growth-inhibiting properties of grifolin and neogrifolin motivated researchers to investigate the underlying mode of cell death in several cancer cell lines [60]. Ye et al., postulated that grifolin induces cell death in nasopharyngeal carcinoma cell line CNE1 through a caspase-mediated apoptosis process [61]. Furthermore, Che et al., investigated the role of autophagy in grifolin-treated human ovarian cancer cells [62]. The research of Che and coworkers suggested that autophagic cell death is induced by grifolin in human ovarian cancer cells by inhibiting the Akt/mTOR/S6K pathway. However, many other grifolin derivatives are still underinvestigated. Especially, the influence of adding or subtracting one or more isoprene units to or from, respectively, the polyisoprene chain of grifolin (**1**)—resulting in compounds **4**, **5**, and **6**—on the anticancer properties is not yet investigated. Therefore, further advanced studies should be conducted to investigate in more detail both the compounds’ potential as anticancer treatments and the safety of these compounds in non-cancer treatments in humans. All the more, since the latter aspect, the impact of those compounds on normal, i.e., healthy cells was not investigated so far.

To the best of our knowledge, this is the first report of the anthelmintic activity of constituents from *Albatrellus confluens* against *C. elegans* and the first investigation of the isolated grifolin (**1**) and neogrifolin (**2**) against the parasitic organisms *S.mansoni* (adult and NTS, and the larval stages of *N. americanus*, *S. ratti*, *H. polygyrus*, and *A. ceylanicum*. It is also the first time the effect of prenyl chain length on the anticancer activity of grifolin derivatives, compounds **4, 5**, and **6,** is investigated.

## 4. Materials and Methods

### 4.1. General

Column chromatography for fractionations or purifications was performed on, silica gel (0.040–0.063 mm, Merck, Darmstadt, Germany). Analytical TLC was performed on pre-coated silica gel F_254_ aluminum sheets (Merck, Darmstadt, Germany) using the solvent system *n*-hexan-EtOAc (3:1) the solvent system and spots were detected by their color, their absorbance under UV-light (254 nm and 366 nm), or after spraying with vanillin and heating. 

Alumina (activated, basic, Brockmann I) was purchased from Sigma-Aldrich (Darmstadt, Germany). Orcinol and prenyl alcohols prenol, geraniol, farnesol, and geranylgeraniol were purchased from Sigma-Aldrich (Darmstadt, Germany). Solvents were purchased from Roth (Karlsruhe, Germany), reagent grade, and used without further purification. 

NMR spectra were recorded with an Agilent DD2 400 MHz NMR spectrometer (Varian, Palo Alto, CA, USA) operating at a proton NMR frequency of 400 MHz using a 5-mm inverse detection cryoprobe. A 2D NMR spectra were recorded using standard CHEMPACK 8.1 pulse sequences (^1^H,^1^H zTOCSY, ^1^H,^13^C gHSQCAD, ^1^H,^13^C gHMBCAD) implemented in Varian VNMRJ 4.2 spectrometer software (Varian, Palo Alto, CA, USA). The mixing time for the TOCSY experiments was set to 80 msec. The HSQC experiment was optimized for ^1^JCH = 146 Hz with DEPT-like editing and ^13^C-decoupling during acquisition time. The HMBC experiment was optimized for a long-range coupling of 8 Hz; a two-step ^1^JCH filter was used (130–165 Hz). ^1^H chemical shifts are referenced to internal TMS (^1^H δ = 0 ppm), while ^13^C chemical shifts are referenced to CDCl_3_ (^13^C δ = 77.0 ppm).

The negative ion-electron spray ionization high-resolution mass spectra (ESI-HRMS) were obtained from an API 3200 Triple Quadrupole System (Sciex, Framingham, MA, USA) equipped with a turbo ion spray source, which performs ionization with an ion spray voltage on 70 eV. Sample introduction was performed by direct injection through an Agilent-HPLC 1200 (Agilent, Santa Clara, CA, USA) syringe pump. During the measurement, the mass/charge range from 5 to 1800 can be scanned.

### 4.2. Fungal Material

The collected fungal fruiting bodies were stored at −20 °C in a refrigerator: *Albatrellus confluens* (Alb. & Schwein.) Kotl. & Pouzar, under *Pinus* sp., Paintner Forst near Kelheim, Bavaria, Germany (8 October 2019, leg./det. N. Arnold, coll. 22/2019); *Albatrellus subrubescens* Kotl. & Pouzar, under *Abies* sp. and *Picea* sp., Paintner Forst near Kelheim, Bavaria, Germany (18 October 2019, leg./det. N. Arnold, coll. 15/2019); *Caloboletus calopus* Pers., under *Fagus* sp., near Gungolding, Bavaria, Germany (Oktober 2019, (leg./det. N. Arnold); *Rhodocollybia maculata* (Alb. & Schwein.) P. Kumm., under *Picea* sp., Reisberg near Ingolstadt, Bavaria, Germany (8 October 2019, leg./det. N. Arnold, coll. 10/2019); *Cortinarius alboviolaceus*, under *Picea* sp., near Hormersdorf, Bavaria, Germany (8 October 2019, leg./det. N. Arnold, coll. 12/2019); *Cortinarius infractus* (Pers.) Fr., under *Picea* sp., near Hormersdorf, Bavaria, Germany (8 October 2019, leg./det. N. Arnold, coll 3/2019); *Cortinarius vulpinus* (Velen.) R. Henry, under *Fagus* sp., Paintner Forst near Kelheim, Bavaria, Germany (8 October 2019, leg./det. N. Arnold, coll. 16/2019); *Hygrophoropsis aurantiaca*, Pegnitz, unde*r Picea* sp., Pegnitz, Veldensteiner Forst near Wilpark Hufeisen, Bavaria, Germany (28 November 2020, leg./det. N. Arnold, coll. 61/2020); *Paralepista flaccida* (Sowerby) Pat., unde*r Picea* sp. and *Fagus* sp., Pegnitz, Veldensteiner Forst near Wilpark Hufeisen, Bavaria, Germany (28 November 2020, leg./det. N. Arnold, coll. 60/2020); *Clitocybe nebularis* (Batsch) Harmaja, under *Pinus* sp., Pegnitz, Veldensteiner Forst near Wilpark Hufeisen, Bavaria, Germany (28 November 2020, leg./det. N. Arnold, coll. 59/2020); *Suillus tridentinus* (Bres.) Singer, under *Larix* sp., Reisberg near Ingolstadt, Bavaria, Germany (18 October 2019, leg./det. N. Arnold, coll. 23/2019). Voucher specimens are deposited at Leibniz-Institute of Plant Biochemistry.

### 4.3. Extract Preparations and Preliminary Anthelmintic Screening

Frozen fungal fruiting bodies (1 g) of each species were macerated and extracted by sonication three times for 15 min with 5 mL of 80% MeOH at room temperature. The resulting solutions were evaporated to dryness under reduced pressure using a rotary evaporator maintained at 40 °C to afford the crude extracts. From each crude extract, a stock solution of 1 mg/mL in 4% DMSO was prepared. The samples were screened for anthelmintic properties against *C. elegans* at the final concentration of 500 µg/mL as described previously [30]. Ivermectin (10 μg/mL) was used as a positive control.

### 4.4. Isolation of Compounds **1** and **2**

Frozen fungal fruiting bodies of *Albatrellus confluens* (375 g) were macerated and extracted three times by sonication for 15 mins with 80% MeOH (3 × 400 mL). The slight yellow solution was evaporated to dryness under reduced pressure using a rotary evaporator maintained at 40 °C to afford 11.6 g of crude extract. The crude extract was redissolved in 100 mL of water and partitioned between *n*-hexane (200 mL × 2), chloroform (200 mL × 2), EtOAc (200 mL × 2), and *n*-butanol (200 mL × 2). The resulting fractions were evaporated to dryness at 40 °C to yield 3.6 g of *n*-hexane, 1.6 g of chloroform, 0.3 g of EtOAc, 1.2 g of *n*-BuOH, and 4.7 g of the remaining aqueous fractions. 

The *n*-hexane, chloroform and EtOAc fractions were combined based on their TLC profiles to give 5.5 g of combined fractions which was adsorbed on an equivalent mass of silica gel and chromatographed over a silica gel column (7 × 34 cm) using *n*-hexane-EtOAc and EtOAc-MeOH gradients as eluents. The column was monitored by UV lamp (254 and 366 nm). Fractions of 35 mL were collected as follows: [(1–6), *n*-hexane-EtOAc (90:10)] [(7–24), *n*-hexane-EtOAc (8:2)], [(25–29), *n*-hexane-EtOAc (7:3)], [(30), *n*-hexane-EtOAc (6:4)], [(31), *n*-hexane-EtOAc (1:1)], [(32), *n*-hexane-EtOAc (4:6)], [(33), *n*-hexane-EtOAc (3:7)], [(34), *n*-hexane-EtOAc (2:8)] [(35), *n*-hexane-EtOAc (1:9)], [(36), EtOAc-MeOH (9:1)] [(37), MeOH (100%)]. These fractions were pooled according to their TLC profiles into 10 subfractions F1 to F10 as follows: F1 (1–3; 0.05 g), F2 (4–7; 2.13 g), F3 (8–10; 0.24 g), F4 (11–13; 0.04 g), F5 (14–18; 0.04 g), F6 (19–23; 1.96 g), F7 (24–28; 0.39 g), F8 (29–31; 0.12 g), F9 (32–35; 0.06 g), and F10 (36–37; 0.39 g). 1.5 g of F2 was separated by silica gel column chromatography (7 × 34 cm) and using *n*-hexane-EtOAc 9:1 as eluent system. A total of 10 fractions of 25 mL each were collected and fractions 4 to 7 were combined to yield compound **1** (767 mg). 1.5 g of F6 was separated by column chromatography using silica gel column chromatography (7 × 34 cm) and *n*-hexane-EtOAc 4:1 as eluent system. A total of 14 fractions of 25 mL each were collected and fractions 5 to 9 were combined to give compound **2** (843 mg). 

### 4.5. Synthesis of **1**, **4**–**6**

Compounds **1**, **4**–**6** were synthesized as described in the literature [35]. Briefly, in each reaction to a solution of orcinol (**3**, 2 g, 16 mmol) the corresponding prenyl alcohol (**7**, 0.55 mL, 5 mmol; **8**, 0.94, 5 mmol; **9**, 1.35 mL, 5 mmol; **10**, 1.75 mL, 5 mmol) in 20 mL dichlormethane acidic alumionia (4 g) was added. In each synthesis, the reaction was refluxed at 60 °C for one week and monitored by TLC plates using vanillin as a spray reagent. After one week, the reaction was cooled down and the reaction mixture was filtered through Celite plug. The filter cake was rinsed with ethyl acetate (500 mL). The ethyl acetate phase was dried with brine and concentrated in vacuo. The crude extract from each reaction was separated by column chromatography on silica gel using *n*-hexane-EtOAc (10:1) as isocratic eluent system and afforded compounds **4** (R*_f_* 0.75, 295 mg, 28.1%), **5** (R*_f_* 0.80, 424 mg, 30.4%), **1** (R*_f_* 0.83, 629 mg, 35.5%), and **6** (R*_f_* 0.86, 819 mg, 38.2%).

### 4.6. In Vitro Anthelmintic Bioassay

#### 4.6.1. *Caenorhabditis elegans*

In the in vitro anthelmintic assay, the Bristol N2 wild-type strain of *C. elegans* was used. The nematodes were cultured on NGM (Nematode Growth Media) Petri plates using the uracil auxotroph *E. coli* strain OP50 as a food source according to the methods described by Stiernagle [63]. The bioassay using *C. elegans* was carried out following the method described earlier [64]. Briefly, an NGM plate containing a 4-day-old *C. elegans* culture was used to harvest worms by washing the plate twice with 2 mL of M9 buffer. The worm suspension was centrifuged at 800 g and resuspended in 2 mL of M9 buffer. The number of worms was adjusted to between 30–40 worms per 20 µL. Using a 384 microtiter well plate 20 µL of the worm suspension was incubated with 20 µL of the test substance for 30 minutes after which the number of living and dead worms in each well was enumerated using a microscope (Olympus BX 41, Tokyo, Japan). In vitro, anthelmintic activity was expressed as the percentage of dead worms. The solvent DMSO (2%) and the standard anthelmintic drug ivermectin (10 μg/mL) were used as negative and positive controls, respectively. All assays were carried out in triplicate and LC_50_ values were calculated using SigmaPlot 14.0.

#### 4.6.2. Parasitic Helminths

The drug sensitivity assays with *S. mansoni* [adult and newly transformed schistosomules (NTS)] and *S. ratti*, *H.s polygyrus*, *N. americanus*, and *A. duodenale* (L3 larvae) to test compounds **1**, **2**, **4**–**10** were carried out in triplicates and conducted as described in previous publications [30,65,66]. All in vitro studies were carried out in accordance with Swiss national and cantonal regulations on animal welfare under permission number 2070 at the Swiss Tropical and Public Health Institute (Swiss TPH). 

### 4.7. Cytotoxic Effects on Human Cancer Cell Lines

The investigated cell lines, PC-3 (human prostate adenocarcinoma) and HT-29 (human colorectal adenocarcinoma) were purchased from ATCC (Manassas, VA, USA). The cell culture medium RPMI 1640, the supplements FCS and l-glutamine, as well as PBS and trypsin/EDTA were purchased from Capricorn Scientific GmbH (Ebsdorfergrund, Germany). Culture flasks, multi-well plates, and further cell culture plastics were purchased from Greiner Bio-One GmbH (Frickenhausen, Germany) and TPP (Trasadingen, Switzerland), respectively. Anti-proliferative and cytotoxic effects, respectively, of the compounds, were investigated by performing colorimetric MTT (3-(4,5-dimethylthiazol-2-yl)-2,5-diphenyltetrazolium bromide) and CV (crystal violet)-based cell viability assays (Sigma-Aldrich, Taufkirchen, Germany), respectively.

#### 4.7.1. Cell Culture

Two human cancer cell lines, PC-3 (prostate adenocarcinoma) and HT-29 (colorectal adenocarcinoma) were used to study cytotoxic and anti-proliferative effects, respectively, of the compounds **1**, **2**, **4**, **5,** and **6**. Both cell lines were cultured in RPMI 1640 medium, supplemented with 10% heat-inactivated FCS, 2 mM l-glutamine, and 1% penicillin/streptomycin, in a humidified atmosphere with 5% CO_2_ at 37 °C. Routinely, cells were cultured in T-75 flasks until reaching subconfluency (~80%). Subsequently, the adherent cells were harvested by washing once with PBS and detaching from plastics by using trypsin/EDTA (0.05% in PBS), prior to cell passaging and seeding for sub-culturing and assays in 96-well plates, respectively.

#### 4.7.2. Cytoxicity Assay

Cells handling and assay techniques were in accordance to the methods described by Khan et al. [67]. In brief, the anti-proliferative and cytotoxic effects of the five compounds under investigation were examined by performing colorimetric MTT (3-(4,5-dimethylthiazol-2-yl)-2,5-diphenyltetrazolium bromide) metabolic cell viability assays and CV (crystal violet)-based cytotoxicity assays, respectively. For this purpose, cells were seeded in low densities in 96-well plates (6000 cells/100 µL/well for PC-3 and 10,000 cells/100 µL/well for HT-29) using the aforementioned cell culture medium. The cells were allowed to adhere for 24 h, followed by the 48 h compound treatment. Based on 20 mM DMSO stock solutions, the compounds **1**, **2**, **4**, **5**, and **6** were serially diluted in standard growth medium to reach final concentrations of 100, 50, 25, 12.5, 6.25, 3.125, 1.56, and 0.78 µM for cell treatment. Furthermore, two controls were used for data normalization (i) negative control (0.5% DMSO) and (ii) positive control (100 µM digitonin) to determine the 100% and 0% viability, respectively. Each data point was determined in independent biological triplicates, each with technical quadruplicates. As soon as the 48 h incubation was finished, MTT and CV assays were conducted.

For the MTT assay, cells were washed once with PBS, followed by incubation with MTT working solution (0.5 mg/mL MTT in culture medium) for 1h under standard growth conditions. After discarding the MTT solution, DMSO was added in order to dissolve the formed formazan, followed by measuring formazan absorbance at 570 nm, and additionally at the reference/background wavelength of 670 nm, by using a SpectraMax M5 multi-well plate reader (Molecular Devices, San Jose, CA, USA).

For the CV assay, cells were washed once with PBS and fixed with 4% paraformaldehyde (PFA) for 20 min at room temperature (RT). After discarding the PFA solution, the cells were left to dry for 10 min and then stained with 1% crystal violet solution for 15 min at RT. The cells were washed with water and dried overnight at RT. Afterward, acetic acid (33% in aqua bidest.) was added to the stained cells and the absorbance was measured at 570 nm and 670 nm (reference wavelength) using a SpectraMax M5 multi-well plate reader (Molecular Devices, San Jose, CA, USA). For data analyses, GraphPad Prism version 8.0.2 was used.

## 5. Conclusions

The in vitro anthelmintic activity against *C. elegans* shown by compounds **1** and **2** was a clear demonstration of the structure activity relationship showing that the position of functional groups has an effect on biological activity. There was also a clear correlation between in vitro anthelmintic activity of the prenyl alcohols against *C. elegans* whereby reducing the length of the prenyl chain increased activity. Although no correlation could be made between the structure of the compounds and anthelmintic activity in the in vitro parasitic assays compounds **4** and **6** displayed promising antischistosomal activity. Compound **6** was also the most promising when the cytotoxic and anti-proliferative effects were investigated. Based on our promising bioactivity for compounds **1**–**2**, **4**–**6**, the synthesis of farnesyl phenols is still an area of interest and should be pursued [68]. Even though prenyl compounds are said to have limited chemical developability they are still enticing scaffolds for the generation of derivatives with the ability to interfere with essential pathways in target organisms like disrupting the transfer or synthesis of essential aliphatic prenyl groups to protein [69].

## Figures and Tables

**Table 1:** In vitro anthelmintic activity of compounds **1**–**10** at 500 µg/mL in % mortality.

Natural and Synthetic Compounds	1	2	3	4	5	6
Anthelmintic activity against *C. elegans*	32.0 ± 4.8	100 ± 0 *	0.7 ± 0.9	85.6 ± 5.1 *	95.3 ± 2.8 *	1.6 ± 2.3
**Corresponding alcohols**	**9**	**9**	**-**	**7**	**8**	**10**
Anthelmintic activity against *C. elegans*	32.1 ± 3.0	32.1 ± 3.0	-	17.8 ± 1.7	99.5 ± 0.7 *	0

* Compound activities determined to be significant versus control worms using Kruskal–Wallis non-parametric test (Statsdirect software version 3.2.8) with *p* < 0.05.

**Table 2:** In vitro anthelmintic activity of compounds **1**–**6** against parasitic helminths.

	Activity % *					
Organism	1	2	4	5	6	Reference
NTS ** (10 µM)	36.0 ± 4	26.0 ± 2	93.3 ± 0 ***	55.0 ± 5.0 ***	75.0 ± 5.0 ***	Auranofin 100
NTS ** (1 µM)	nt	nt	38.9 ± 1.7	nt	55.0 ± 1.7 ***	nt
*S. mansoni* (10 µM)	27.3 ± 2	35.1 ± 2	29.2 ± 4.2	0	16.7 ± 0	Praziquantel 100
*N. americanus* (10 µM)	16.5 ± 12.5	13.4 ± 8.4	12.1 ± 8.9	29.6 ± 2.4	28.2 ± 7.4	Levamisole 100
*S. ratti* (10 µM)	18.6 ± 7.8	18 ± 2	1.9 ± 13.1	2.9 ± 5.6	0	Levamisole 100
*H. polygyrus* (10 µM)	16.4 ± 0.4	16 ± 4.3	23.4 ± 7.3	38.6 ± 4.8	36.6 ± 1.9	Levamisole 100
*A. ceylanicum* (10 µM)	2.0 ± 2.5	7.5 ± 0	24.4 ± 7.8	19 ± 14.1	17.5 ± 3.9	Abamectin 100

* Mortality % based on three replicates; ** NTS = newly transformed schistosomula; nt = not tested. *** compound activities determined to be significant versus control worms using Kruskal–Wallis non-parametric test (Statsdirect software version 3.2.8) with *p* < 0.05.

**Table 3:** In vitro anthelmintic activity of prenyl alcohols **7**–**10** against parasitic helminths.

	Activity % *				
Organism	7	8	9	10	Reference
NTS ** (10 µM)	33.3 ± 0	31.3 ± 2	25 ± 0	31.3 ± 2	Auranofin 100
*S. mansoni* (10 µM)	nt	nt	nt	nt	nt
*N. americanus* (10 µM)	11.6 ± 2	11.3 ± 10	21.3 ± 1	36.3 ± 1	Levamisole 100
*S. ratti* (10 µM)	13 ± 5	39.6 ± 0.4	0.2 ± 9	0	Levamisole 100
*H. polygyrus* (10 µM)	0	1.8 ± 5	34.6 ± 5.5	0	Levamisole 100
*A. ceylanicum* (10µM)	18.8 ± 2.9	13.7 ± 1.7	10 ± 6.4	23.6 ± 5.9	Abamectin 100

* Mortality % based on three replicates; ** NTS = newly transformed schistosomula; nt = not tested.

## Data Availability

All spectroscopic data of isolated compounds are reported in the Supplementary Materials.

## Supplementary Materials

The following supporting information can be downloaded at: https://www.mdpi.com/article/10.3390/molecules27092950/s1, Figures S1–S6: ESI-HRMS, 1D and 2D NMR of compound **1**, Table S1. ^1^H (400 MHz) and ^13^C (100 MHz) NMR data of compound **1** in CDCl_3_; Figures S7–S13: ESI-HRMS, 1D and 2D NMR of compound **2**, Table S2. ^1^H (400 MHz) and ^13^C (100 MHz) NMR data of compound **2** in CDCl_3_; Figures S14–S22: HRMS, 1D and 2D NMR of compound **4**, Table S3. ^1^H (400 MHz) and ^13^C (100 MHz) NMR data of compound **4** in CDCl_3_; Figures S23–S25: HRMS, ^1^H NMR spectrum and ^13^C NMR of compound **5**, Table S4. ^1^H (400 MHz) and ^13^C (100 MHz) NMR data of compound **5** in CDCl_3_; Figures S26–S33: HRMS, 1D and 2D NMR of compound **6**, Table S5. ^1^H (400 MHz) and ^13^C (100 MHz) NMR data of compound **6** in CDCl_3_; Figure S34 LC50 curve for in vitro anthelmintic activity of compound **2** (neogrifolin) against *C. elegans*.

## References

[1] Moser W., Schindler C., Keiser J. (2019). Drug combinations against soil transmitted helminth infections. Adv. Parasitol..

[2] Spangenberg T. (2021). Alternatives to Praziquantel for the prevention and control of schistosomiasis. ACS Infect. Dis..

[3] Sarkodie S.A., Owusu P.A. (2021). Global assessment of environment, health and economic impact of the novel coronavirus (COVID-19). Environ. Dev. Sustain..

[4] Brooker S.J., Ziumbe K., Negussu N., Crowley S., Hammami M. (2021). Neglected tropical disease control in a world with COVID-19: An opportunity and a necessity for innovation. Trans. R. Soc. Trop. Med. Hyg..

[5] Ehrenberg J.P., Utzinger J., Fontes G., da Rocha E.M.M., Ehrenberg N., Zhou X.-N., Steinmann P. (2021). Efforts to mitigate the economic impact of the COVID-19 pandemic: Potential entry points for neglected tropical diseases. Infect. Dis. Poverty.

[6] Mooney D., Richards K.G., Danaher M., Grant J., Gill L., Mellander P.E., Coxon C.E. (2021). An analysis of the spatio-temporal occurrence of anthelmintic veterinary drug residues in groundwater. Sci. Total Environ..

[7] Pérez-Cogollo L.C., Rodríguez-Vivas R.I., del Socorro Basto-Estrella G., Reyes-Novelo E., Martinez-Morales I., Ojeda-Chi M.M., Favila M.E. (2018). Toxicity and adverse effects of macrocyclic lactones on dung beetles: A review. Rev. Mex. Biodivers..

[8] Wit J., Dilks C.M., Andersen E.C. (2021). Complementary approaches with free-living and parasitic nematodes to understanding anthelmintic resistance. Trends Parasitol..

[9] Sharpton T.J., Combrink L., Arnold H.K., Gaulke C.A., Kent M. (2020). Harnessing the gut microbiome in the fight against anthelminthic drug resistance. Curr. Opin. Microbiol..

[10] Tinkler S.H. (2020). Preventive chemotherapy and anthelmintic resistance of soil transmitted helminths—Can we learn nothing from veterinary medicine?. One Health.

[11] Crellen T., Walker M., Lamberton P.H., Kabatereine N.B., Tukahebwa E.M., Cotton J.A., Webster J.P. (2016). Reduced efficacy of praziquantel against *Schistosoma mansoni* is associated with multiple rounds of mass drug administration. Clin. Infect. Dis..

[12] Walker M., Freitas L.T., Halder J.B., Brack M., Keiser J., King C.H., Levecke B., Lim Y.A., Pieri O., Sow D. (2022). Improving anthelmintic treatment for schistosomiasis and soil transmitted helminthiases through sharing and reuse of individual participant data [version 1; peer review: Awaiting peer review]. Wellcome Open Res..

[13] Hawksworth D.L., Lücking R., Heitman J., Howlett B.J. (2017). Chapter 4, Fungal diversity revisited: 2.2 to 3.8 million species. The Fungal Kingdom.

[14] Hawksworth D.L. (2001). Mushrooms: The extent of the unexplored potential. Int. J. Med. Mushrooms.

[15] Wasser S.P. (2014). Medicinal mushroom science: Current perspectives, advances, evidences, and challenges. Biomed. J..

[16] Zeb M., Lee C.H. (2021). Medicinal properties and bioactive compounds from wild mushrooms native to North America. Molecules.

[17] Comans-Perez R.J., Sanchez J.E., Al-Ani L.K.T., Gonzalez-Cortazar M., Castaneda-Ramirez G.S., Mendoza de Gives P., Sanchez-Garcia A.D., Millan-Orozco J., Aguilar-Marcelino L. (2020). Biological control of sheep nematode *Haemonchus contortus* using edible mushrooms. Biol. Control.

[18] Sasaki T., Takagi M., Yaguchi T., Miyadoh S., Okada T., Koyama M. (1992). A new anthelmintic cyclodepsipeptide, PF1022A. J. Antibiot..

[19] Harder A., Holden-Dye L., Walker R., Wunderlich F. (2005). Mechanisms of action of emodepside. Parasitol. Res..

[20] Assmus F., Hoglund R.M., Monnot F., Specht S., Scandale I., Tarning J. (2022). Drug development for the treatment of onchocerciasis: Population pharmacokinetic and adverse events modeling of emodepside. PLoS Negl. Trop. Dis..

[21] Thorn R.G., Barron G.L. (1984). Carnivorous mushrooms. Science.

[22] Heydari R., Pourjam E., Goltapeh E.M. (2006). Antagonistic effect of some species of *Pleurotus* on the root-knot nematode*, Meloidogyne javanica* in vitro. Plant Pathol. J..

[23] Pineda-Alegria J.A., Sanchez-Vazquez J.E., Gonzalez-Cortazar M., Zamilpa A., Lopez-Arellano M.E., Cuevas-Padilla E.J., Mendoza-de-Gives P., Aguilar-Marcelino L. (2017). The edible mushroom *Pleurotus djamor* produces metabolites with lethal activity against the parasitic nematode *Haemonchus contortus*. J. Med. Food.

[24] Sreedevi R., Pradeeb B. (2016). V Anthelmintic and antibacterial activity of red pigment from *Aspergillus terreus.* Res. J. Pharm. Biol. Chem. Sci..

[25] Trappe J.M., Claridge A.W., Dighton J., White J.F., Oudemans P. (2005). Hypogeous fungi: Evolution of reproductive and dispersal strategies through interactions with animals and mycorrhizal plants. The Fungal Community.

[26] Spiteller P. (2008). Chemical defence strategies of Higher fungi. Chem. Eur. J..

[27] Anke H., Sterner O. (1997). Nematicidal metabolites from higher fungi. Curr. Org. Chem..

[28] Chitwood D.J. (2002). Phytochemical based strategies for nematode control. Annu. Rev. Phytopathol..

[29] Garcia-Bustos J.F., Sleebs B.E., Gasser R.B. (2019). An appraisal of natural products active against parasitic nematodes of animals. Parasites Vectors.

[30] Dube M., Saoud M., Rennert R., Fotso G.W., Andrae-Marobela K., Imming P., Häberli C., Keiser J., Arnold N. (2021). Anthelmintic activity and cytotoxic effects of compounds isolated from the fruits of *Ozoroa insignis* Del. (Anacardiaceae). Biomolecules.

[31] Ryvarden L., Gilbertson R.L. (1993). European Polypores Part I. Fungiflora.

[32] Koch B., Steglich W. (2007). Monoterpenoid pigments from *Albatrellus fletti* (Basidiomycetes). Eur. J. Org. Chem..

[33] Nukata M., Hashimoto T., Yamamoto I., Iwasaki N., Tanaka M., Asakawa Y. (2002). Neogrifolin derivatives possessing anti-oxidative activity from the mushroom *Albatrellus ovinus*. Phytochemistry.

[34] Yaqoob A., Li W.M., Liu V., Wang C., Mackedenski S., Tackaberry L.E., Massicotte H.B., Egger K.N., Reimer K., Lee C.H. (2020). Grifolin, neogrifolin and confluentin from the terricolous polypore *Albatrellus flettii* suppress KRAS expression in human colon cancer cells. PLoS ONE.

[35] Jentsch N.G., Zhang X., Magolan J. (2020). Efficient synthesis of cannabigerol, grifolin, and piperogalin via alumina-promoted allylation. J. Nat. Prod..

[36] Besl H., Höfle G., Jendrny B., Jägers E., Steglich W. (1977). Pilz pigmente, XXXI. Farnesylphenole aus *Albatrellus*-Arten (*Basidiomycetes*). Chem. Ber..

[37] Zhi-Hui D., Ze-Jun D., Liu J.-K. (2001). Albaconol, A novel prenylated resorcinol (=benzene-1,3-diol) from Basidiomycetes *Albatrellus confluens*. Helv. Chim. Acta.

[38] Yang X.-L., Quin C., Wang F., Dong Z.J., Liu J.-K. (2008). A New meroterpenoid pigment from the Basidiomycete *Albatrellus confluens*. Chem. Biodivers.

[39] Hashimoto T., Quang D.N., Nukada M., Asakawa Y. (2005). Isolation, synthesis and biological activity of grifolic acid dervivatives from the inedible mushroom *Albatrellus dispansus*. Heterocycles.

[40] Guo H., Li Z.-H., Feng T., Liu J.-K. (2015). One new ergostane-type steroid and three new phthalide derivatives from cultures of the basidiomycete *Albatrellus confluens*. J. Asian Nat. Prod. Res..

[41] Hellwig V., Nopper R., Mauler F., Freitag J., Liu J.-K., Ding Z.-H., Stadler M. (2003). Activities of prenylphenol derivatives from fruitbodies of *Albatrellus* spp. on the human and rat vanilloid receptor 1 (VR1) and characterisation of the novel natural product, confluentin. Arch. Der Pharm..

[42] Wang F., Luo D.-Q., Liu J.-K. (2005). Aurovertin E, a new polyene pyrone from the basidiomycete *Albatrellus confluens*. J. Antibiot..

[43] Hirata Y., Nahanishi K. (1950). Grifolin, an antibiotic from a basidiomycete. J. Biol. Chem..

[44] Cardillo G., Cricchio R., Merlini L., Nasini G. (1969). Poliisoprenifoli naturali. Sintesis della grifolina e della ostrutina. Gazz Chim. Ital..

[45] Quang D.N., Hashimoto T., Asakawa Y. (2006). Inedible mushrooms: A good source of biologically active substances. Chem. Rec..

[46] Mahiou V., Roblot F., Hocquemiller R., Cavé A. (1995). Piperogalin, a new prenylated diphenol from *Peperomia galioides*. J. Nat. Prod..

[47] Ali H.M., Abo-Shady A., Sharaf Eldeen H.A., Soror H.A., Shousha W.G., Abdel-Barry O.A., Saleh A.M. (2013). Structural features, kinetics and SAR study of radical scavenging and antioxidant activities of phenolic and anilinic compounds. Chem. Cent. J..

[48] Baiocchi T., Anesko K., Mercado N., Park H., Kin K., Strickhouser-Monzon B., Robles P., Bowman C., Wang H., Sternberg P.W. (2020). Signaling by AWC olfactory neurons is necessary for *Caenorhabditis elegans*’ response to prenol, an odor associated with nematode-infected insects. Genetics.

[49] Kumaran A.M., D’Souza P., Agarwal A., Bokkolla R.M., Balasubramaniam M. (2003). Geraniol, the putative anthelmintic principle of *Cymbopogon martinii*. Phytother. Res..

[50] Otify A.M., Serag A., Porzel A., Wessjohann L.A., Farag M.A. NMR Metabolome-based classification of *Cymbopogon* species: A prospect for phyto-equivalency of its different accessions using chemometric tools. Food Anal. Methods.

[51] Nirmal S.A., Girme A.S., Bhalke R.D. (2007). Major constituents and anthelmintic activity of volatile oils from leaves and flowers of *Cymbopogon martini* Roxb. Nat. Prod. Res..

[52] Jaradat N., Adwan L., K’aibni S., Shraim N., Zaid A.N. (2016). Chemical composition, anthelmintic, antibacterial and antioxidant effects of *Thymus bovei* essential oil. BMC Complement Altern Med..

[53] Abdel-Rahman F.H., Alaniz N.M., Saleh M.A. (2013). Nematicidal activity of terpenoids. J. Environ. Sci. Health B.

[54] Navarro-Moll M.C., Romero M.C., Montilla M.P., Valero A. (2011). In vitro and in vivo activity of three sesquiterpenes against L3 larvae of *Anisakis* type I. Exp. Parasitol..

[55] Menna-Barreto R.F., Laranja G.A., Silva M.C., Coelho M.G., Paes M.C., Oliveira M.M., de Castro S.L. (2008). Anti-Trypanosoma cruzi activity of *Pterodon pubescens* seed oil: Geranylgeraniol as the major bioactive component. Parasitol. Res..

[56] Mirza Z., Soto E.R., Hu Y., Nguyen T.-T., Koch D., Aroian R.V., Ostroff G.R. (2020). Anthelmintic activity of yeast particle-encapsulated terpenes. Molecules.

[57] Michiels J., Missotten J., Dierick N., Fremaut D., Maene P., De Smet S. (2008). In vitro degradation and in vivo passage kinetics of carvacrol, thymol, eugenol and trans-cinnamaldehyde along the gastrointestinal tract of piglets. J. Sci. Food Agric..

[58] Gobert G.N., Tran M.H., Moertel L., Mulvenna J., Jones M.K., McManus D.P., Loukas A. (2010). Transcriptional changes in *Schistosoma mansoni* during early schistosomula development and in the presence of erythrocytes. PLoS Negl. Trop. Dis..

[59] Sirak B., Asres K., Hailu A., Dube M., Arnold N., Häberli C., Keiser J., Imming P. (2021). In Vitro Antileishmanial and antischistosomal activities of anemonin isolated from the fresh leaves of *Ranunculus multifidus* Forsk. Molecules.

[60] Bouyahya A., El Allam A., Zeouk I., Taha D., Zengin G., Goh B.H., Catauro M., Montesano D., El Omari N. (2022). Pharmacological effects of grifolin: Focusing on anticancer mechanisms. Molecules.

[61] Ye M., Liu J.K., Lu Z.X., Zhao Y., Liu S.F., Li L.L., Tan M., Weng X.X., Li W., Cao Y. (2005). Grifolin, a potential antitumor natural product from the mushroom *Albatrellus confluens*, inhibits tumor cell growth by inducing apoptosis in vitro. FEBS Lett..

[62] Che X., Yan H., Sun H., Dongol S., Wang Y., Lv Q., Jiang J. (2016). Grifolin induces autophagic cell death by inhibiting the Akt/mTOR/S6K pathway in human ovarian cancer cells. Oncol. Rep..

[63] Stiernagle T. Maintenance of *C. elegans* (11 February 2006). In WormBook; The *C. elegans* Research Community, Ed. http://www.wormbook.org.

[64] Thomsen H., Reider K., Franke K., Wessjohann L.A., Keiser J., Dagne E., Arnold N. (2012). Characterization of constituents and anthelmintic properties of *Hagenia abyssinica*. Sci. Pharm..

[65] Lombardo F.C., Pasche V., Panic G., Endriss Y., Keiser J. (2019). Life cycle maintenance and drug-sensitivity assays for early drug discovery in *Schistosoma mansoni*. Nat. Protoc..

[66] Keiser J., Haeberli C. (2021). Evaluation of commercially available anthelmintics in laboratory models of human intestinal Nematode infections. ACS Infect. Dis..

[67] Khan M.F., Nasr F.A., Noman O.M., Alyhya N.A., Ali I., Saoud M., Rennert R., Dube M., Hussain W., Green I.R. (2020). Cichorins D–F: Three New Compounds from *Cichorium intybus* and Their Biological Effects. Molecules.

[68] Grabovyi G.A., Mohr J.T. (2016). Total synthesis of grifolin, grifolic acid, LL-Z1272α, LL-Z1272β, and ilicicolinic acid A. Org. Lett..

[69] Zhang F.L., Casey P.J. (1996). Protein prenylation: Molecular mechanisms and functional consequences. Ann. Rev. Biochem..

