# Supplementary material for: Albatrellus confluens (Alb. & Schwein.) Kotl. & Pouz.: Natural Fungal Compounds and Synthetic Derivatives with In Vitro Anthelmintic Activities and Antiproliferative Effects against Two Human Cancer Cell Lines
Source: Molecules. 2022 May 5;27(9):2950. doi: 10.3390/molecules27092950 (PMC9100200; doi:10.3390/molecules27092950)
Supplement: Supplementary file 1 [file molecules-27-02950-s001.zip › molecules-1674611 Supplementary.pdf]

Supplemental data

Albatrellus confluens (Alb. & Schwein.) Kotl. & Pouz. : natural fungal compounds and synthetic derivatives with anthelmintic and anticancer activities

Mthandazo Dube[1], Dayma Llanes[1], Mohamad Saoud[1], Robert Rennert[1], Peter Imming[2], Cécile Häberli[3,4], Jennifer Keiser[3,4] and Norbert Arnold[1*]

[1] Department of Bioorganic Chemistry, Leibniz Institute of Plant Biochemistry, Weinberg 3, D-06120 Halle (Saale), Germany; Mthandazo.Dube@ipb-halle.de (M.D.); Dayma.LlanesMartinez@ipb-halle.de (D.L); Mohamad.Saoud@ipb-halle.de (M.S.); Robert.Rennert@ipb-halle.de (R.R.); Norbert.Arnold@ipb-halle.de (N.A.)

[2] Institute of Pharmacy, Faculty of Natural Sciences, Martin-Luther-University Halle-Wittenberg, D-06120 Halle (Saale), Germany; peter.imming@pharmazie.uni-halle.de (P.I.)

[3] Swiss Tropical and Public Health Institute, Kreuzstr.2, CH-4123 Allschwil, Switzerland; cecile.haeberli@swisstph.ch (C.H.), jennifer.keiser@swisstph.ch (J.K.)

[4] University of Basel, CH-4051 Basel, Switzerland

* Correspondence: Norbert.Arnold@ipb-halle.de (N.A.); Tel.: +49-345-5582-1310 (N.A.)[1]

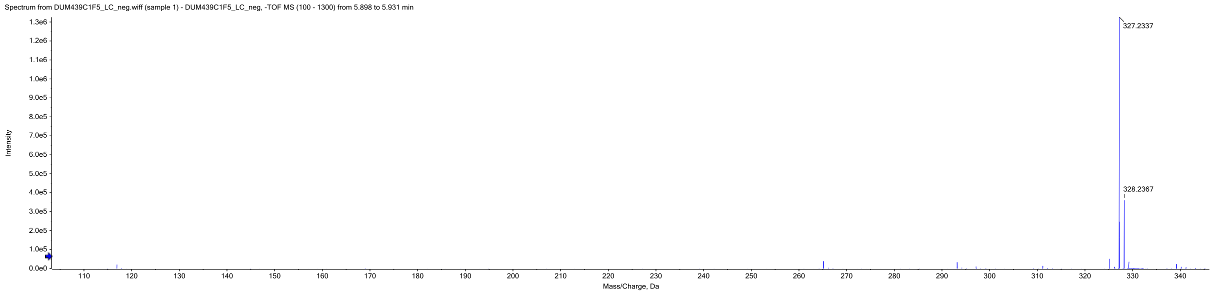

Figure S1: ESI-HRMS (neg. ion mode) spectrum of grifolin (**1**).

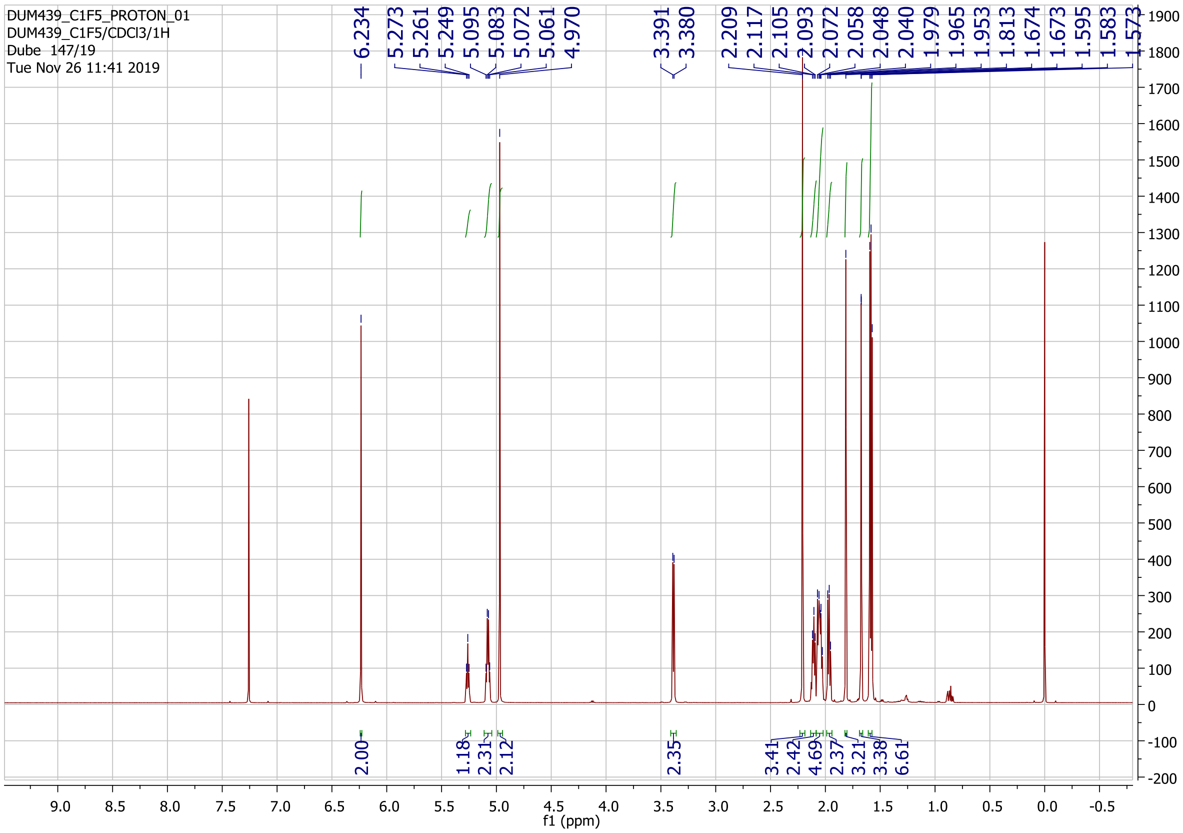

Figure S2: ^{1}H NMR spectrum of grifolin (**1**) in $CDCl_3$ (400 MHz, δ in ppm).

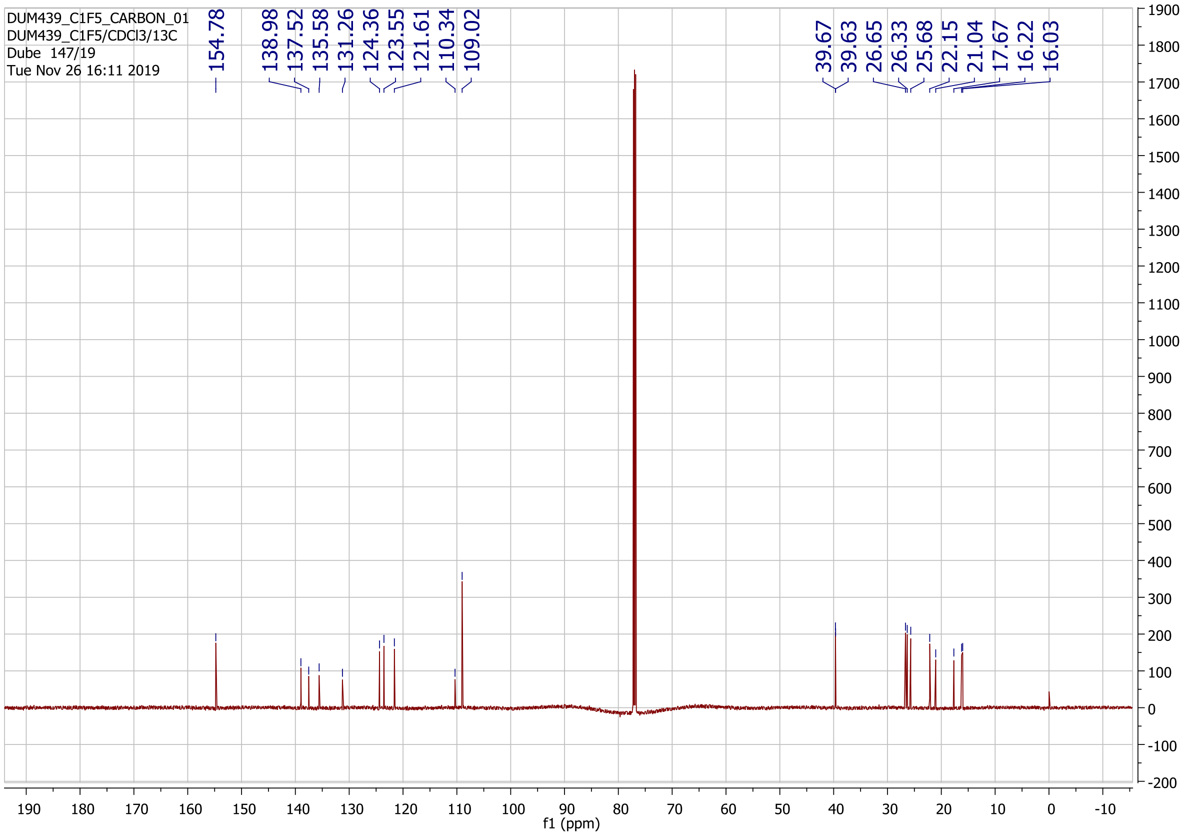

Figure S3: ^{13}C NMR spectrum of grifolin (**1**) in $CDCl_3$ (400 MHz, δ in ppm).

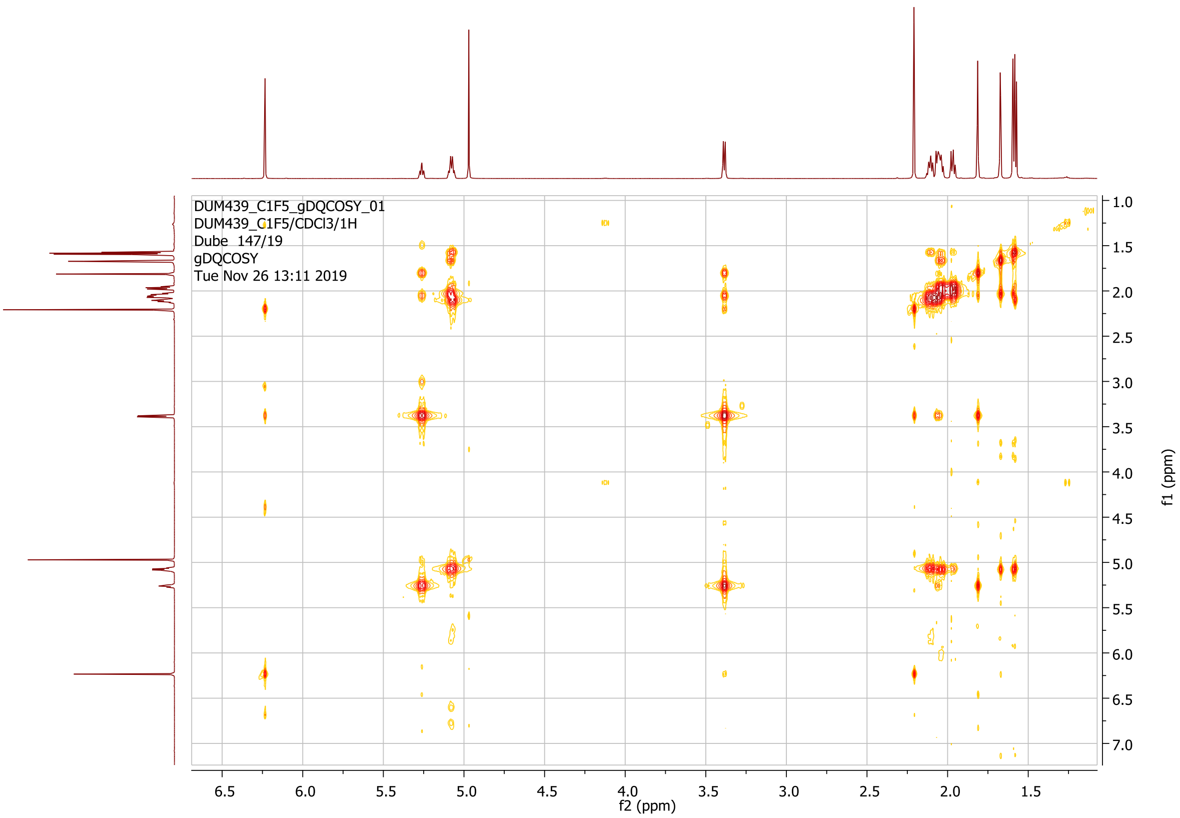

Figure S4: gDQCOSY spectrum of grifolin (**1**) in $CDCl_3$ (400 MHz, δ in ppm).

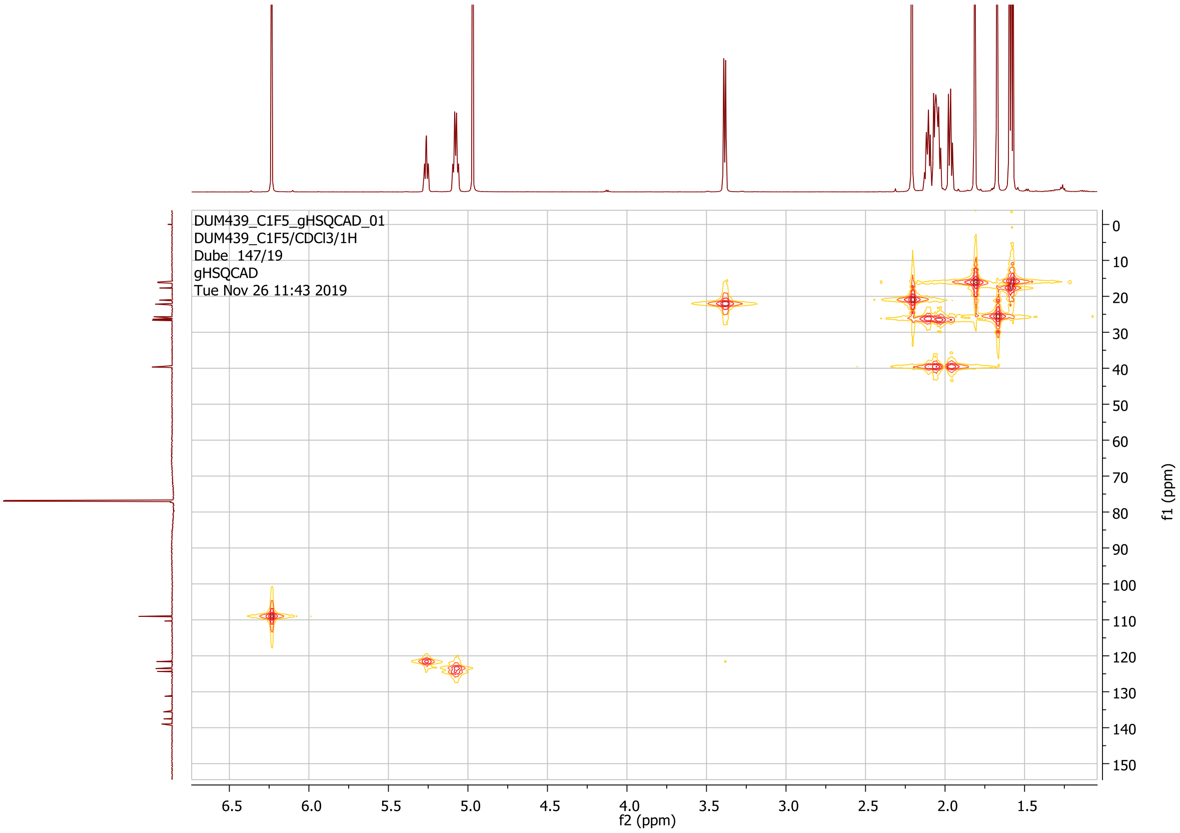

Figure S5: gHSQC spectrum of grifolin (**1**) in $CDCl_3$ (400 MHz, δ in ppm).

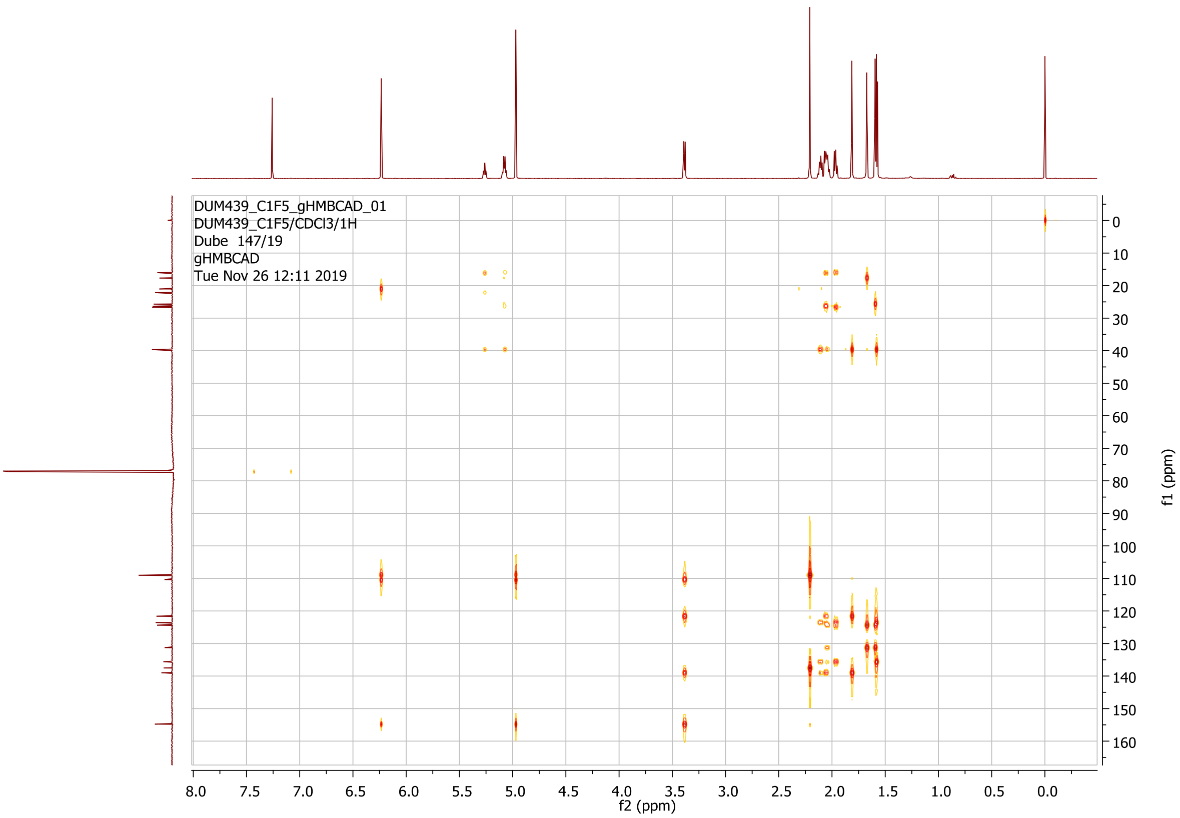

Figure S6: gHMBC spectrum of grifolin (**1**) in $CDCl_3$ (400 MHz, δ in ppm).

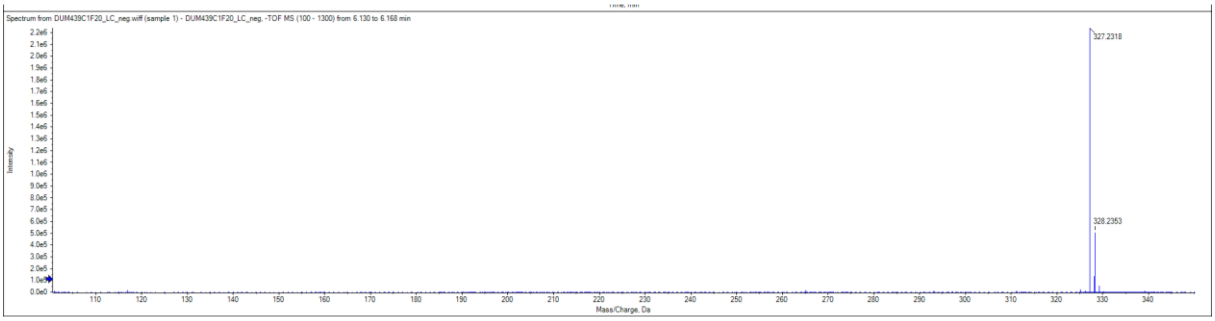

Figure S7: ESI-HRMS (neg. ion mode) of neogrifolin *(***2***)*.

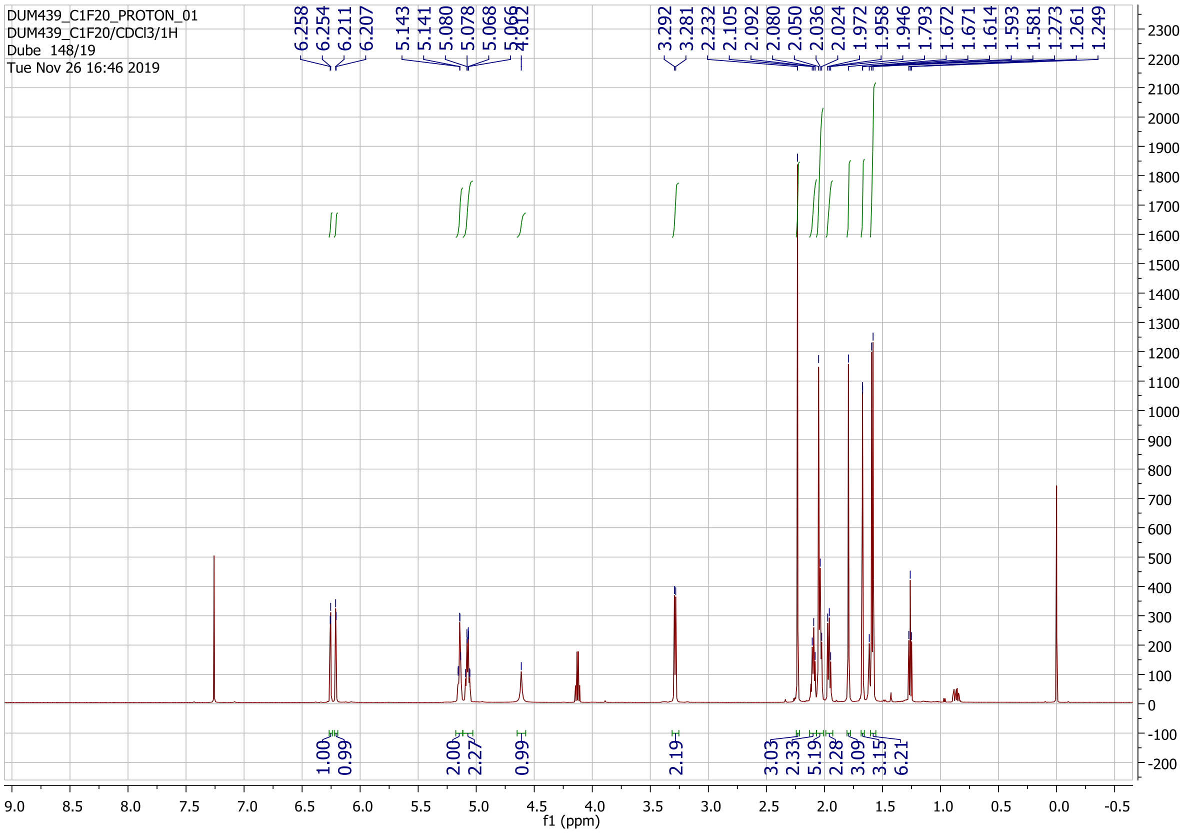

Figure S8: ^{1}H NMR spectrum of neogrifolin (**2**) in $CDCl_3$ (400 MHz, δ in ppm).

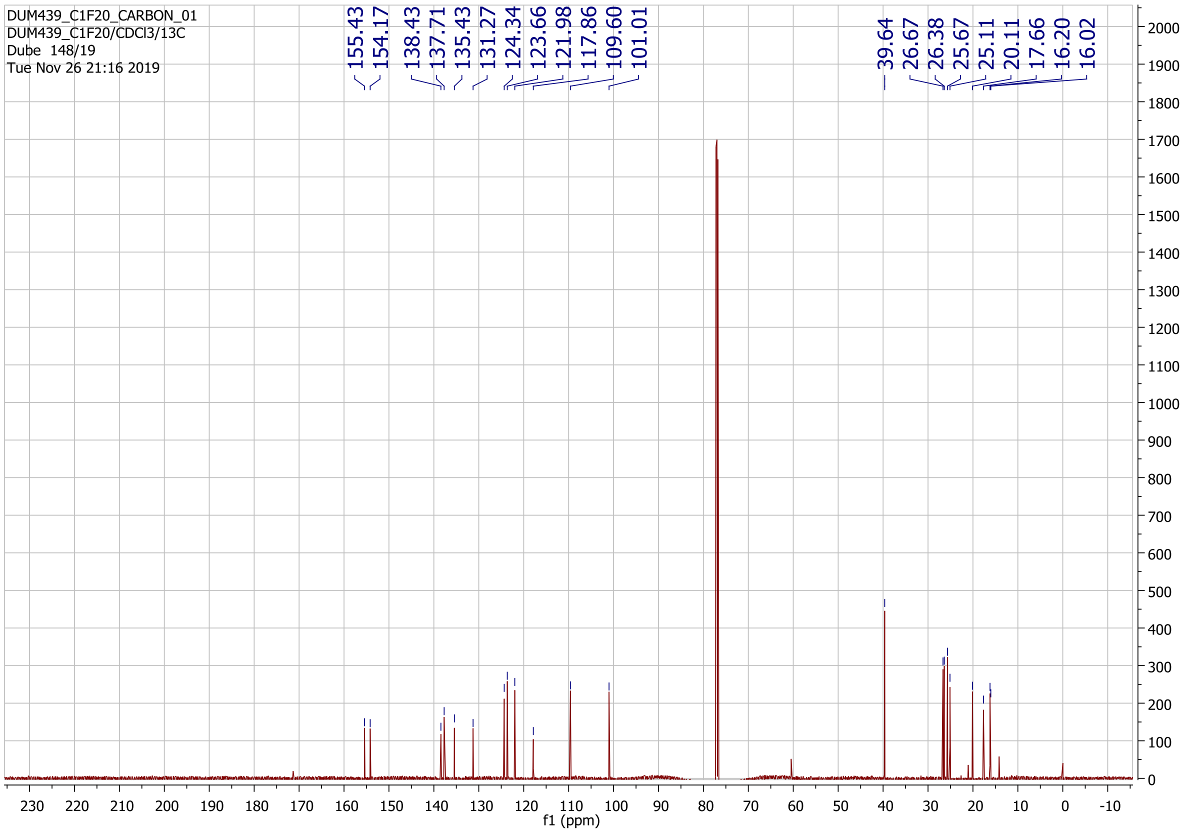

Figure S9: ^{13}C NMR spectrum of neogrifolin (**2**) in $CDCl_3$ (400 MHz, δ in ppm).

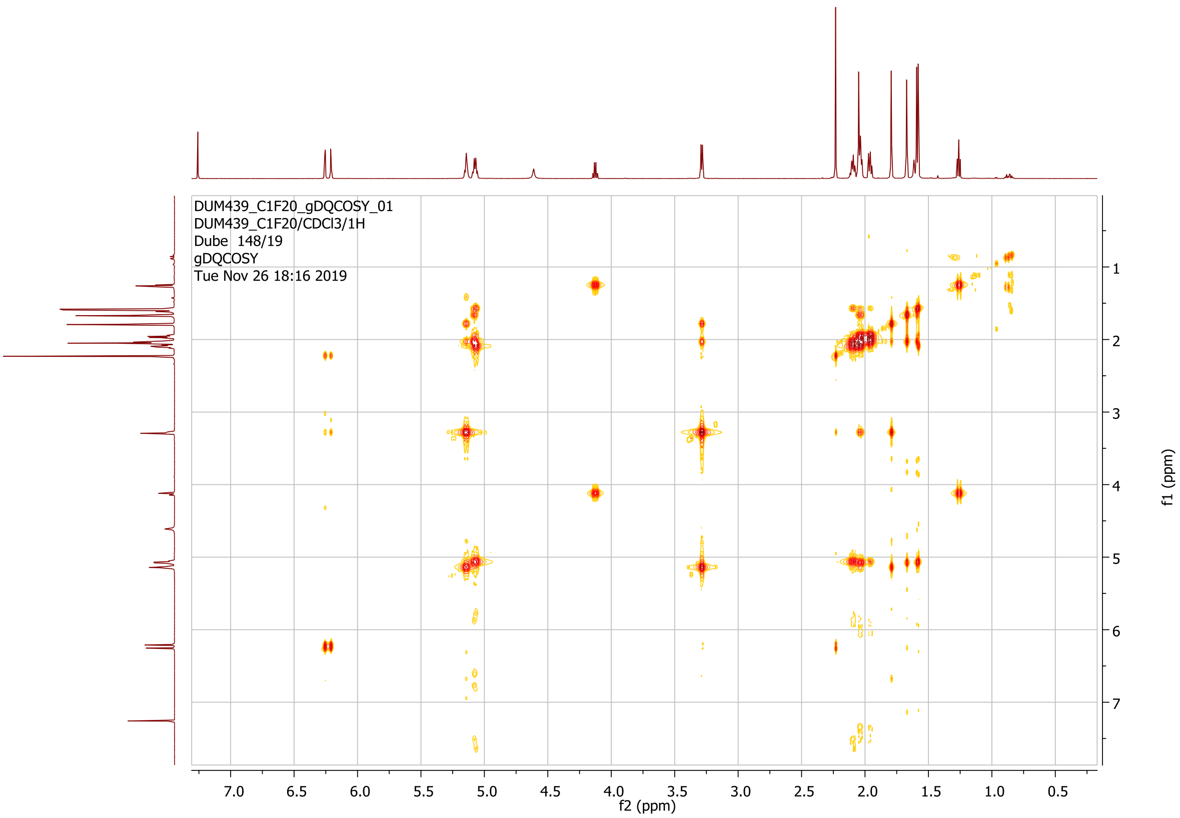

Figure S10: gDQCOSY spectrum of neogrifolin (**2**) in $CDCl_3$ (400 MHz, δ in ppm).

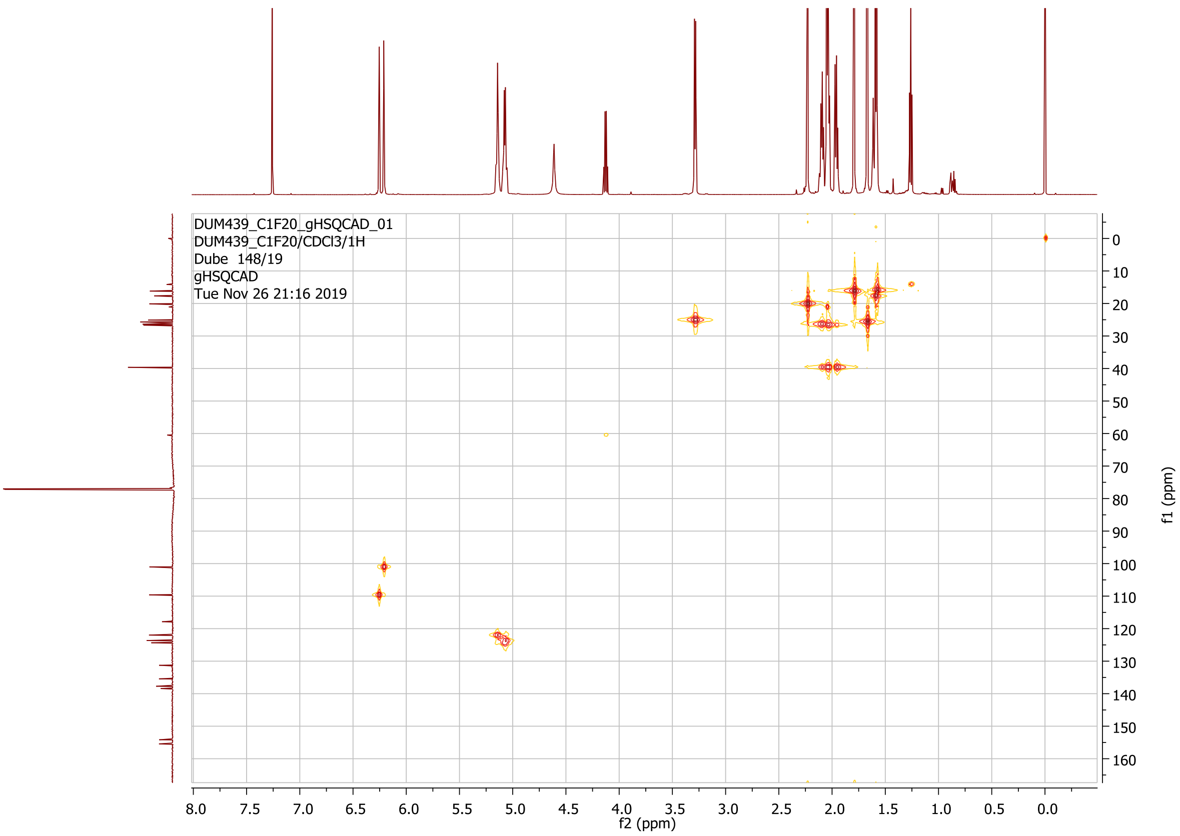

Figure S11: gHSQC spectrum of neogrifolin (**2**) in $CDCl_3$ (400 MHz, δ in ppm).

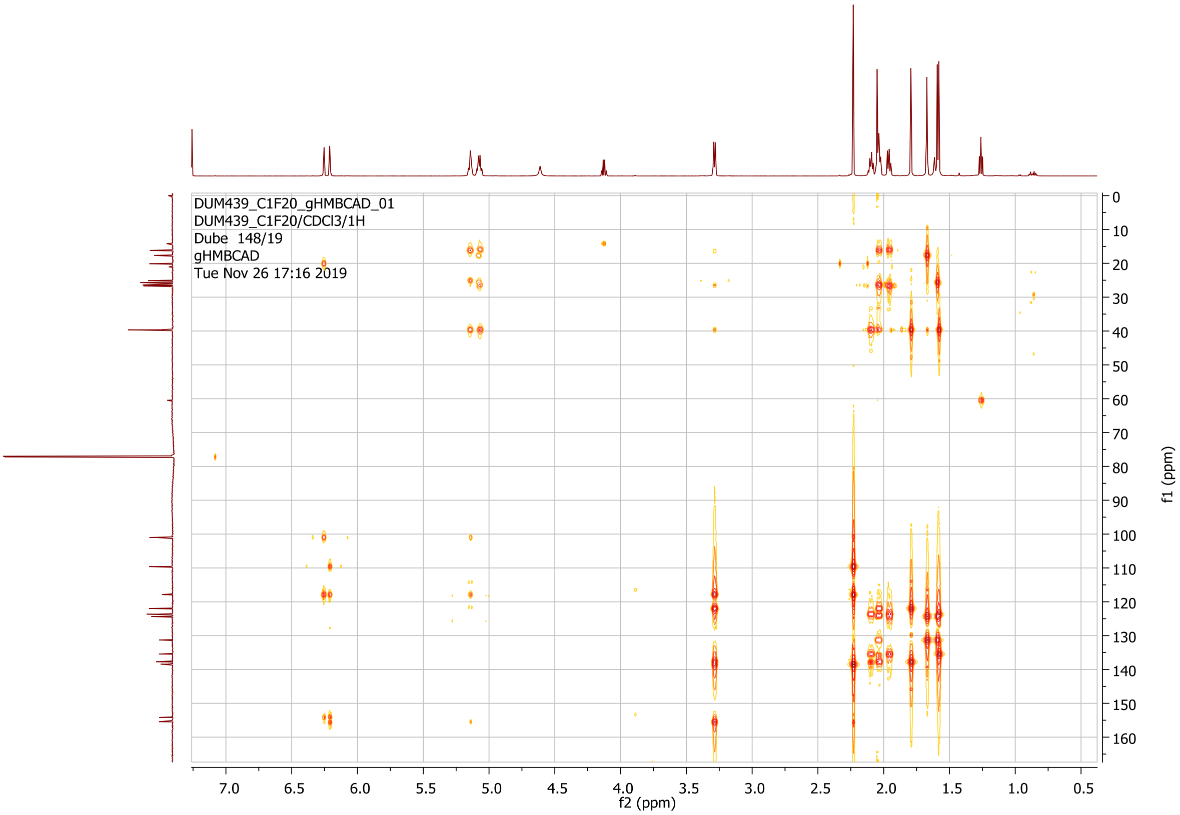

Figure S12: gHMBC spectrum of neogrifolin (**2**) in $CDCl_3$ (400 MHz, δ in ppm).

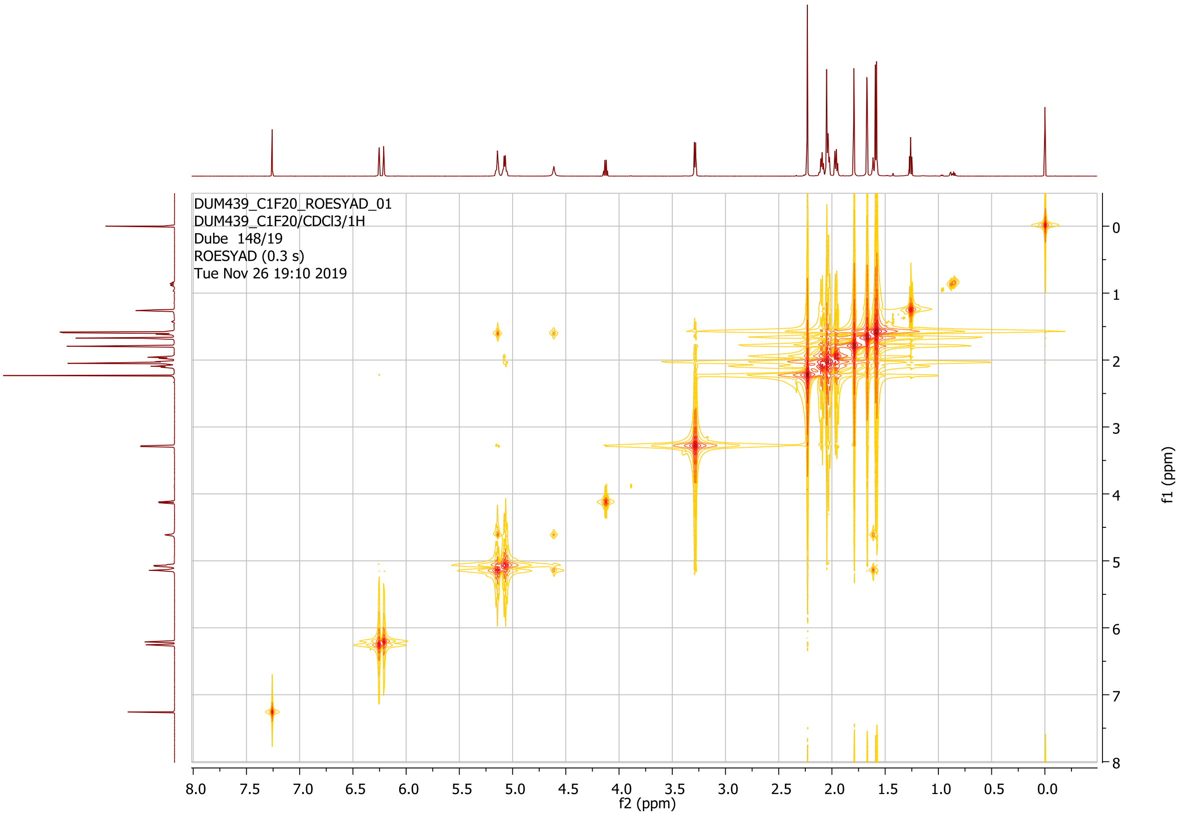

Figure S13: NOESY spectrum of neogrifolin (**2**) in $CDCl_3$ (400 MHz, δ in ppm).

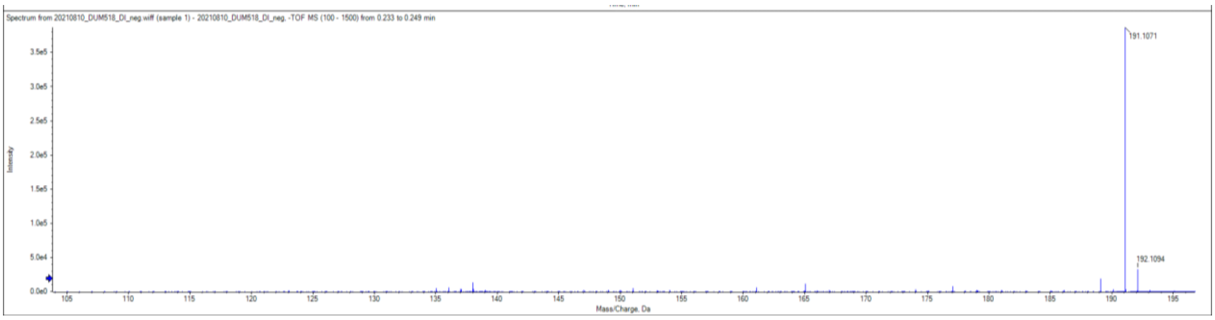

Figure S14: ESI-HRMS (neg. ion mode) of prenyl-2-orcinol (**4**).

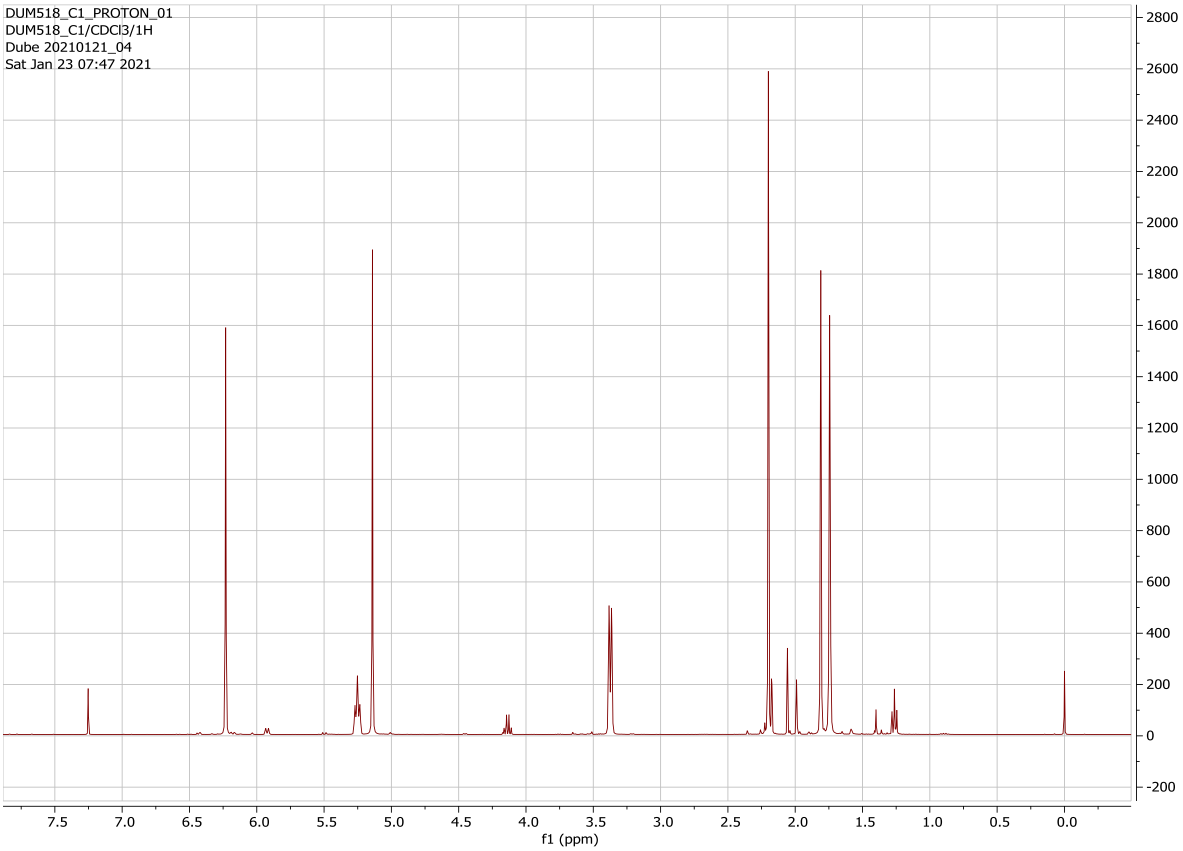

Figure S15: ^{1}H NMR spectrum of prenyl-2-orcinol (**4**) in $CDCl_3$ (400 MHz, δ in ppm) without integration.

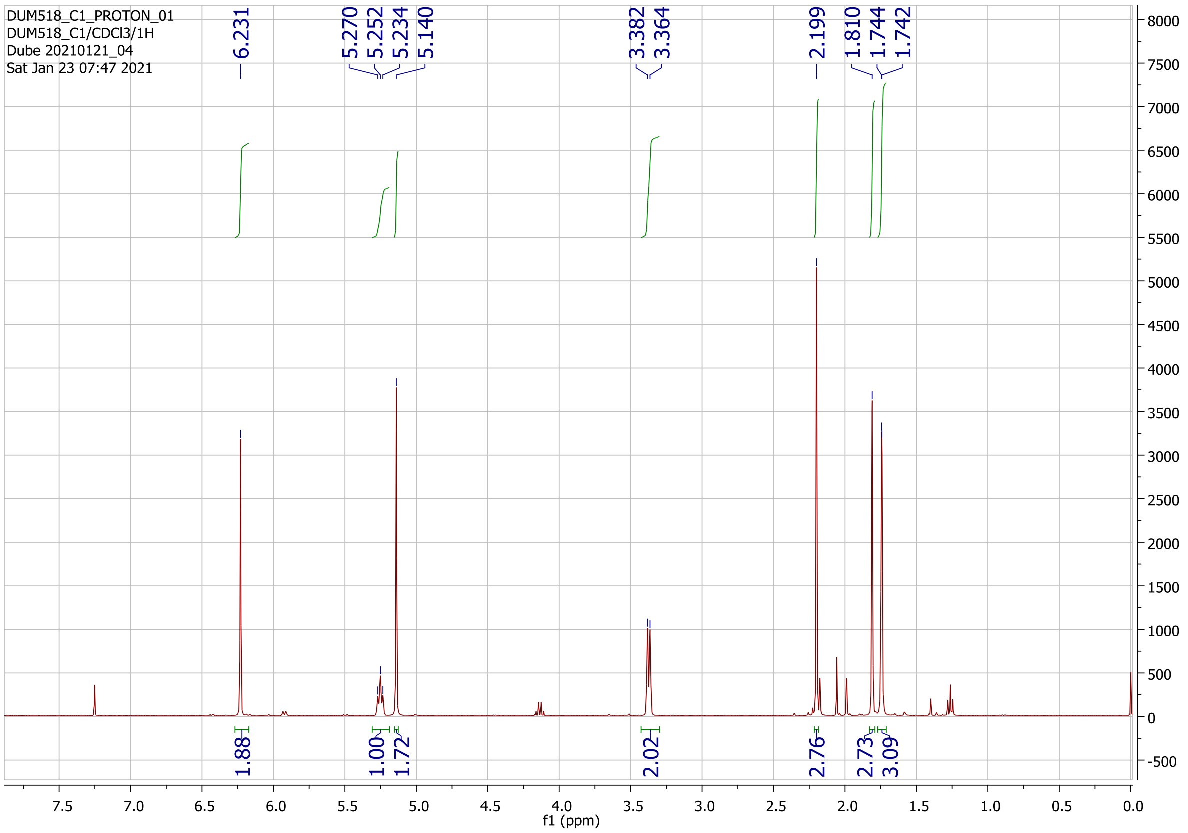

Figure S16: ^{1}H NMR spectrum of prenyl-2-orcinol (**4**) in $CDCl_3$ (400 MHz, δ in ppm).

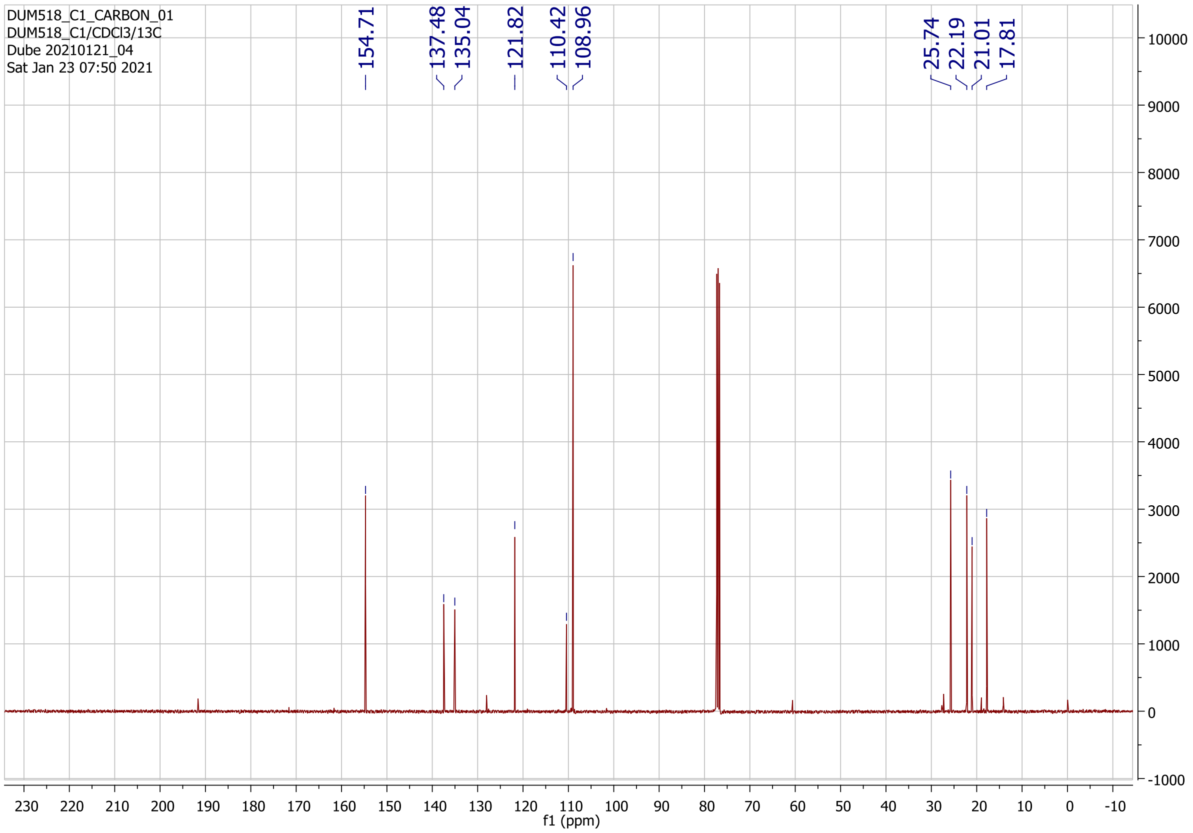

Figure S17: ^{13}C NMR spectrum of prenyl-2-orcinol (**4**) in $CDCl_3$ (400 MHz, δ in ppm).

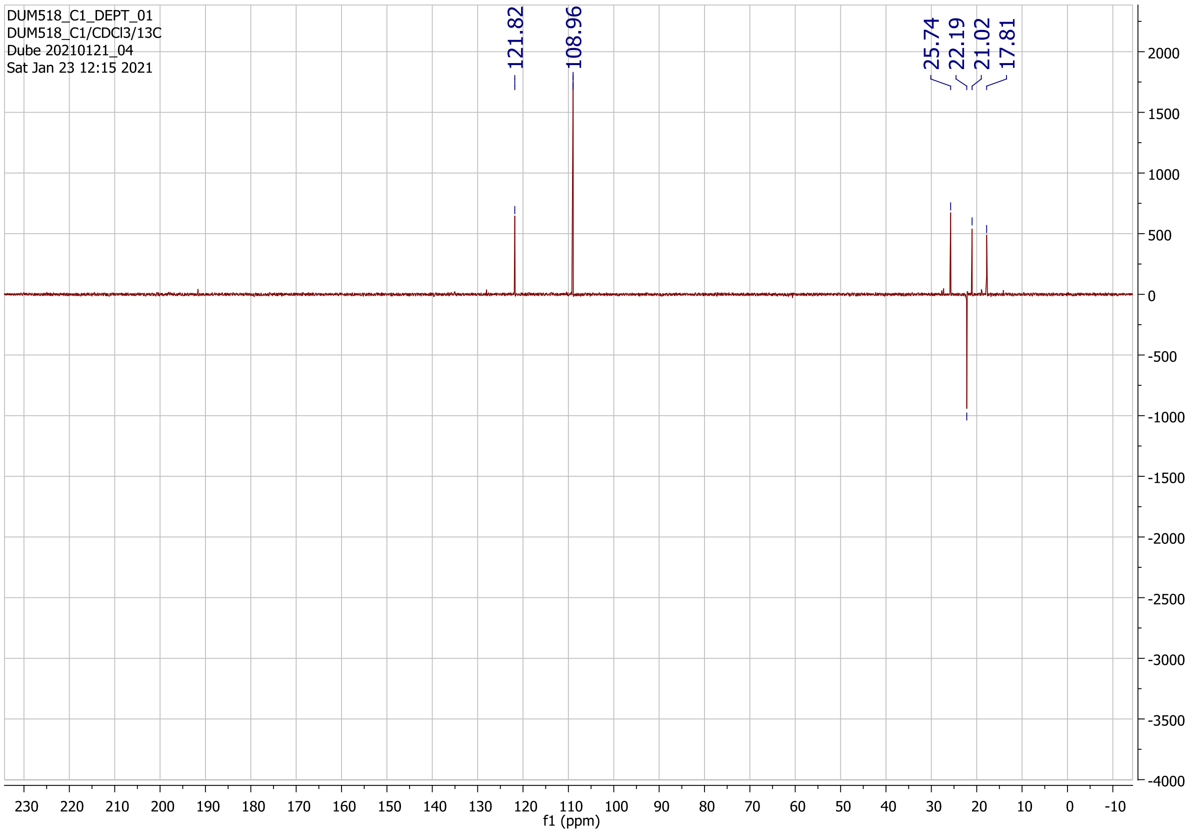

Figure S18: DEPT 135 spectrum of prenyl-2-orcinol (**4**) in $CDCl_3$ (400 MHz, δ in ppm).

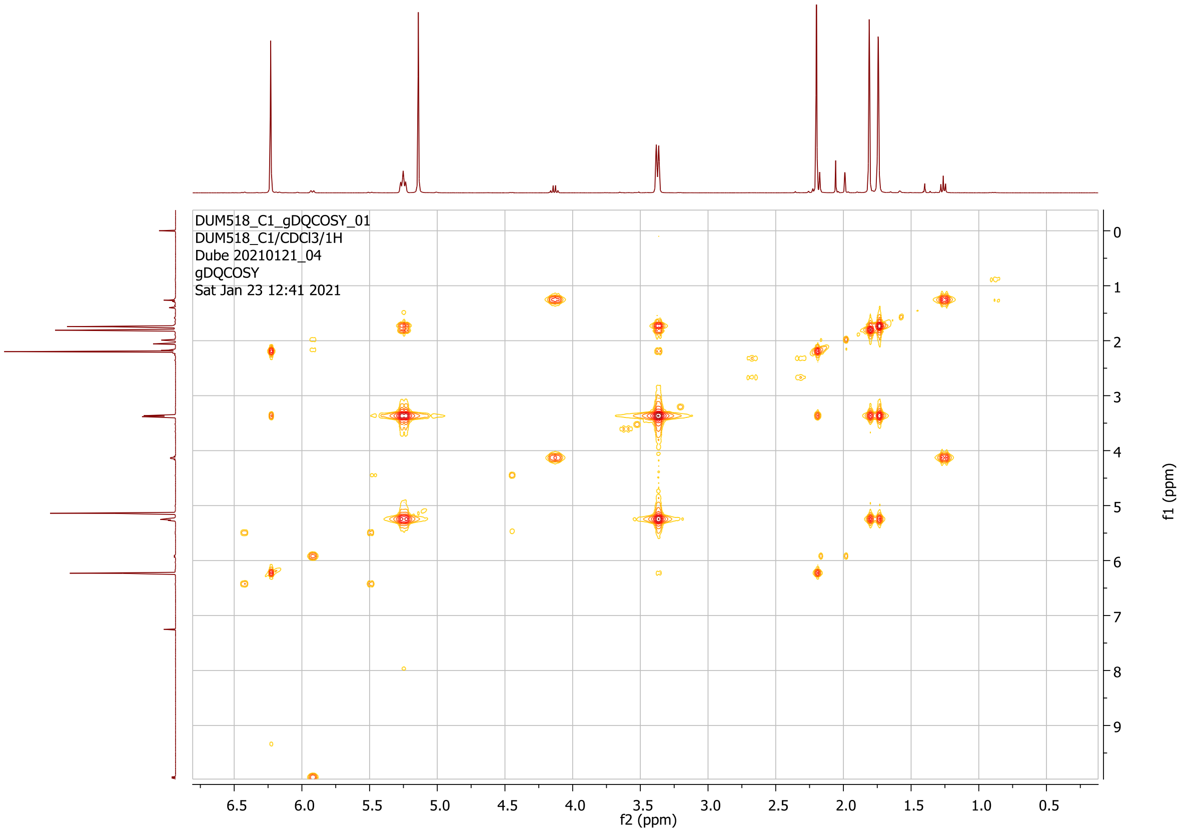

Figure S19: gDQCOSY spectrum of prenyl-2-orcinol (**4**) in $CDCl_3$ (400 MHz, δ in ppm).

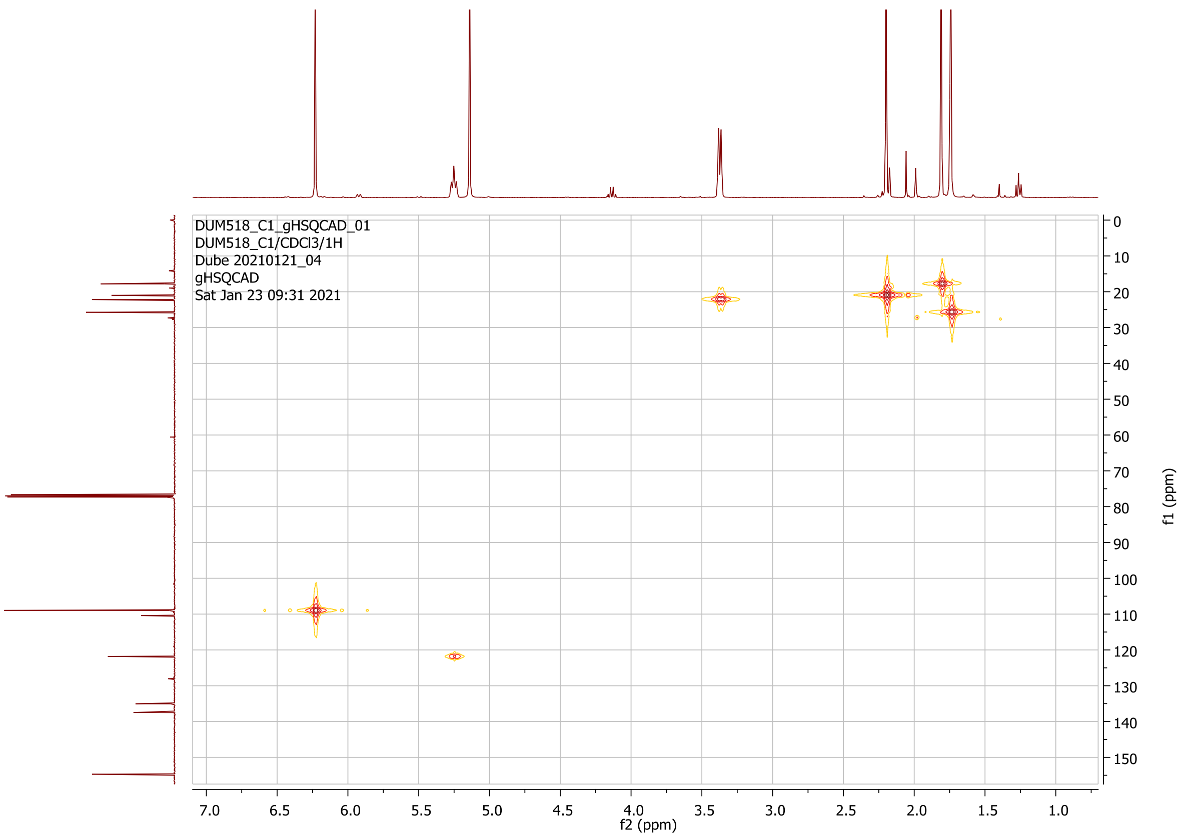

Figure S20: gHSQC spectrum of prenyl-2-orcinol (**4**) in $CDCl_3$ (400 MHz, δ in ppm).

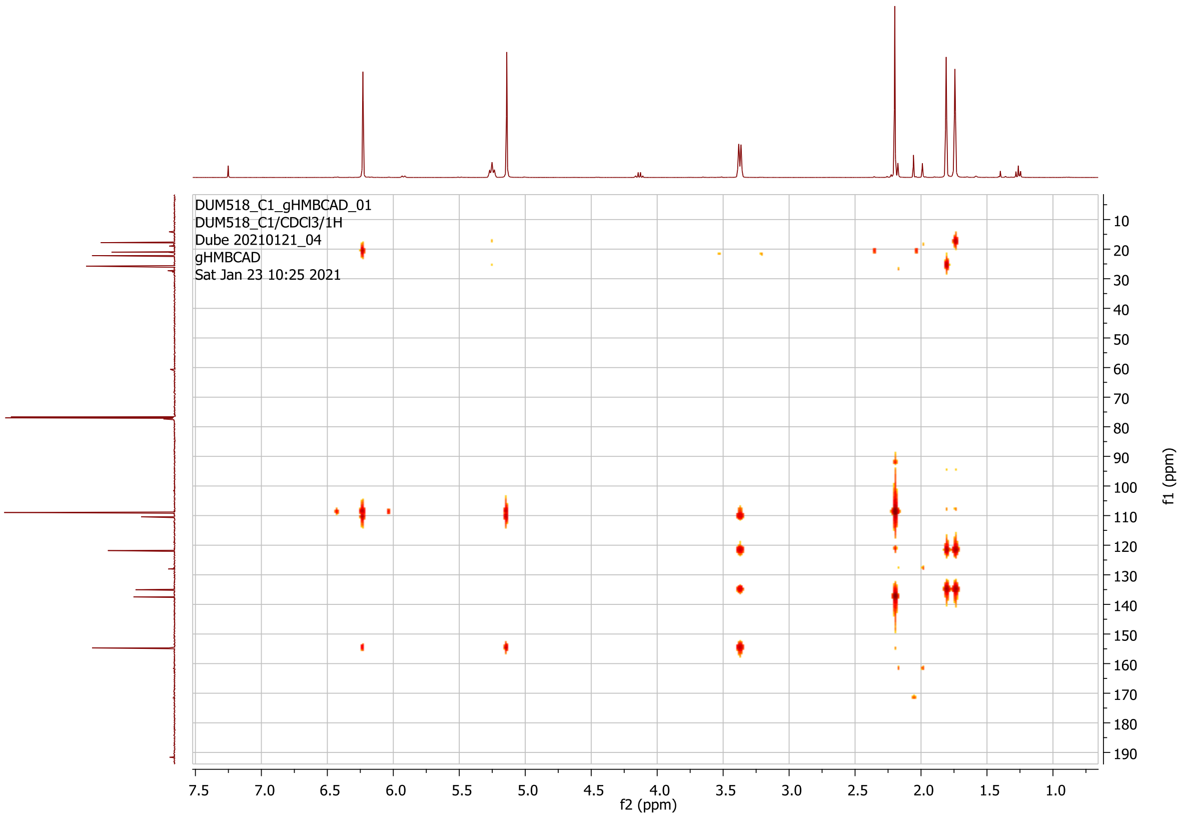

Figure S21: gHMBC spectrum of prenyl-2-orcinol (**4**) in $CDCl_3$ (400 MHz, δ in ppm).

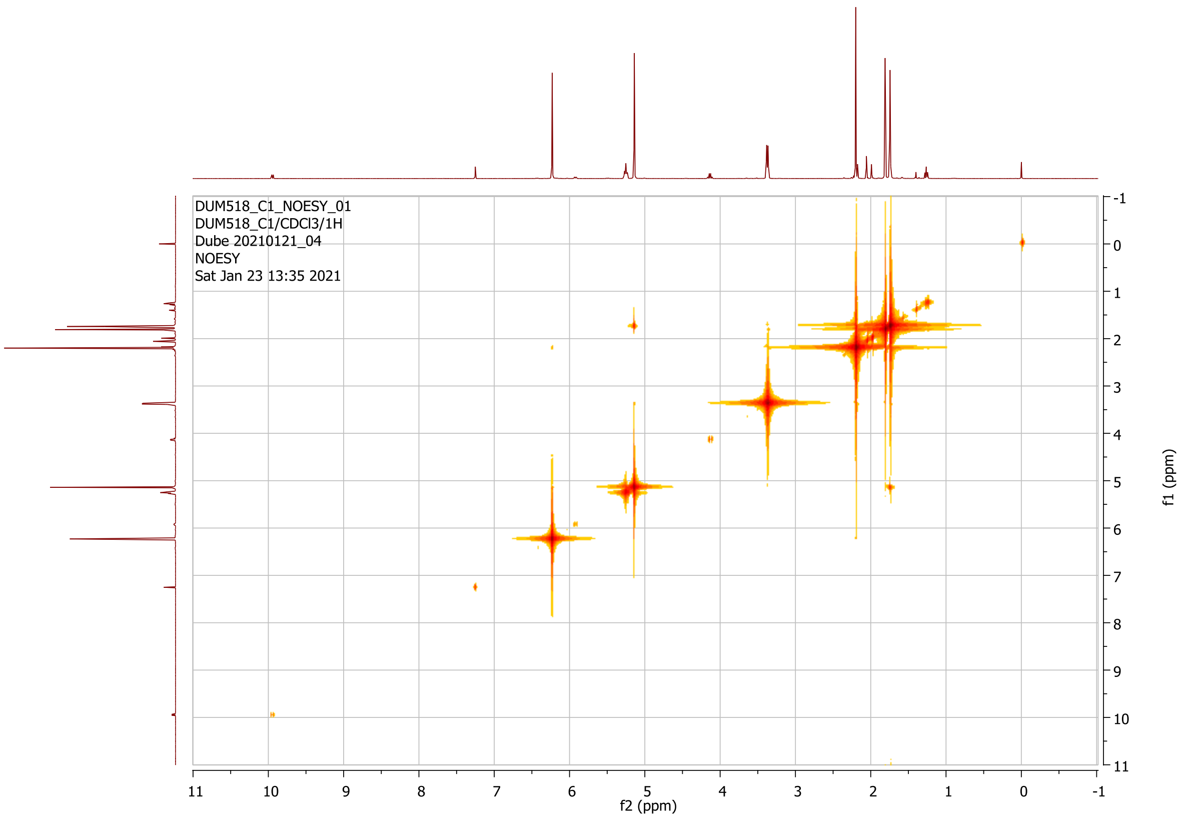

Figure S22: NOESY spectrum of prenyl-2-orcinol (**4**) in CDCl₃ (400 MHz, δ in ppm).

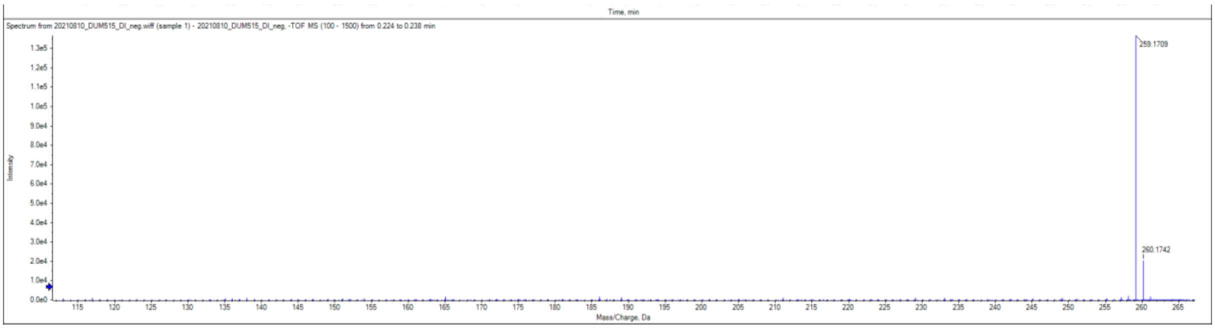

Figure S23: ESI-HRMS (neg. ion mode) spectrum of geranyl-2-orcinol (**5**).

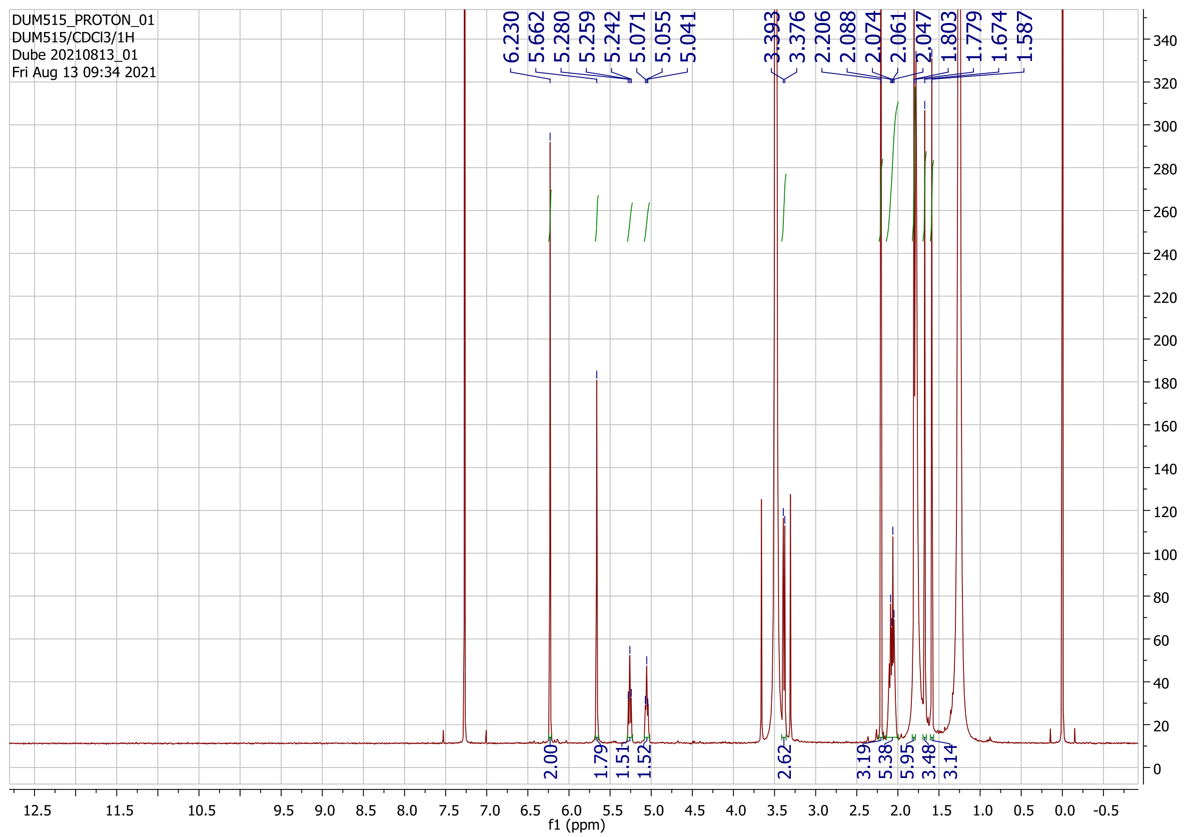

Figure S24: ^{1}H NMR spectrum of geranyl-2-orcinol (**5**) in $CDCl_3$ (400 MHz, δ in ppm).

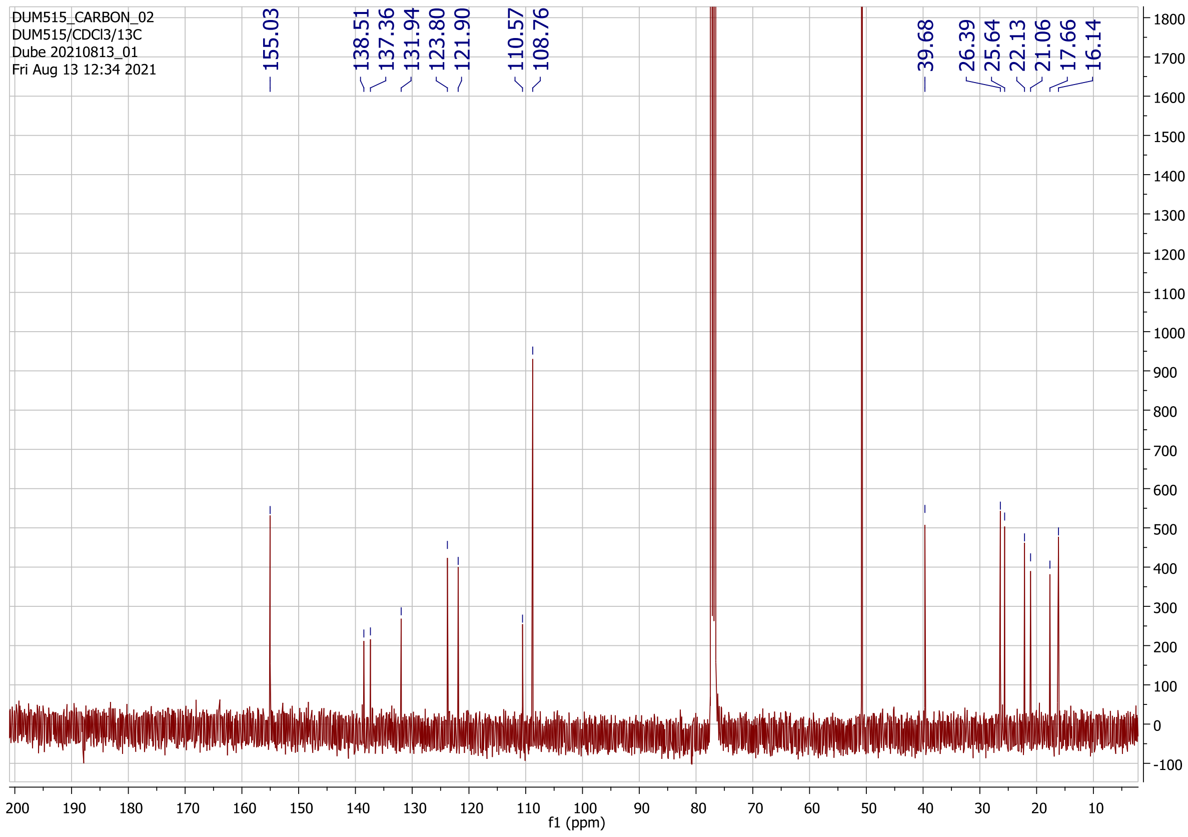

Figure S25: ^{13}C NMR spectrum of geranyl-2-orcinol (**5**) in $CDCl_3$ (400 MHz, δ in ppm).

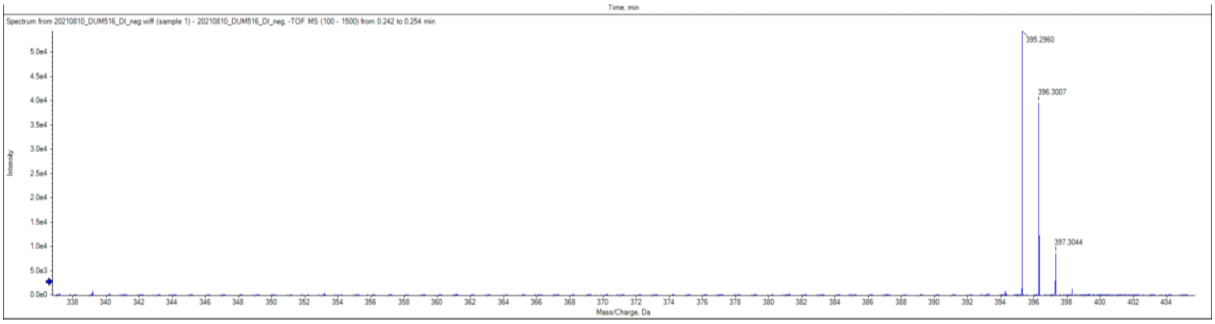

Figure S26: ESI-HRMS (neg. ion mode) spectrum of geranylgeranyl-2-orcinol (**6**).

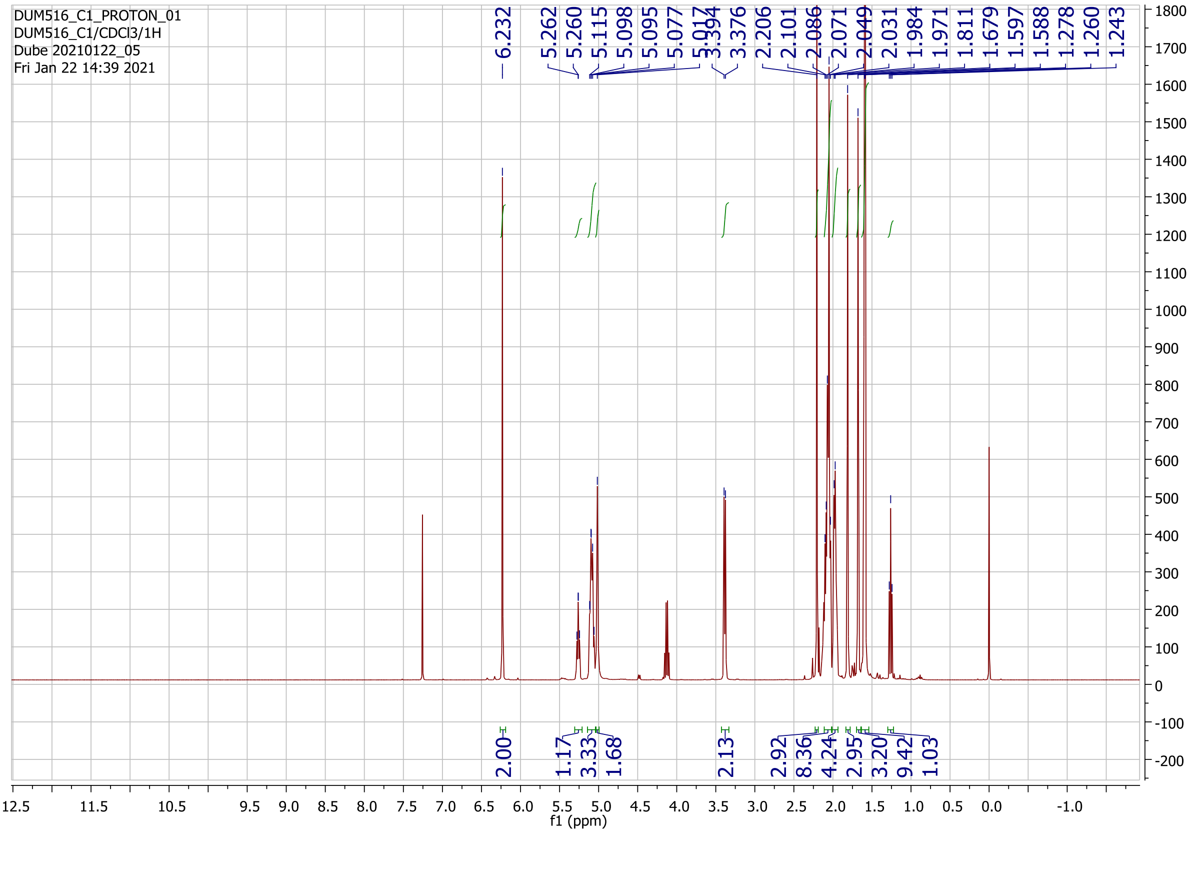

Figure S27: ^{1}H NMR spectrum of geranylgeranyl-2-orcinol (**6**) in $CDCl_3$ (400 MHz, δ in ppm).

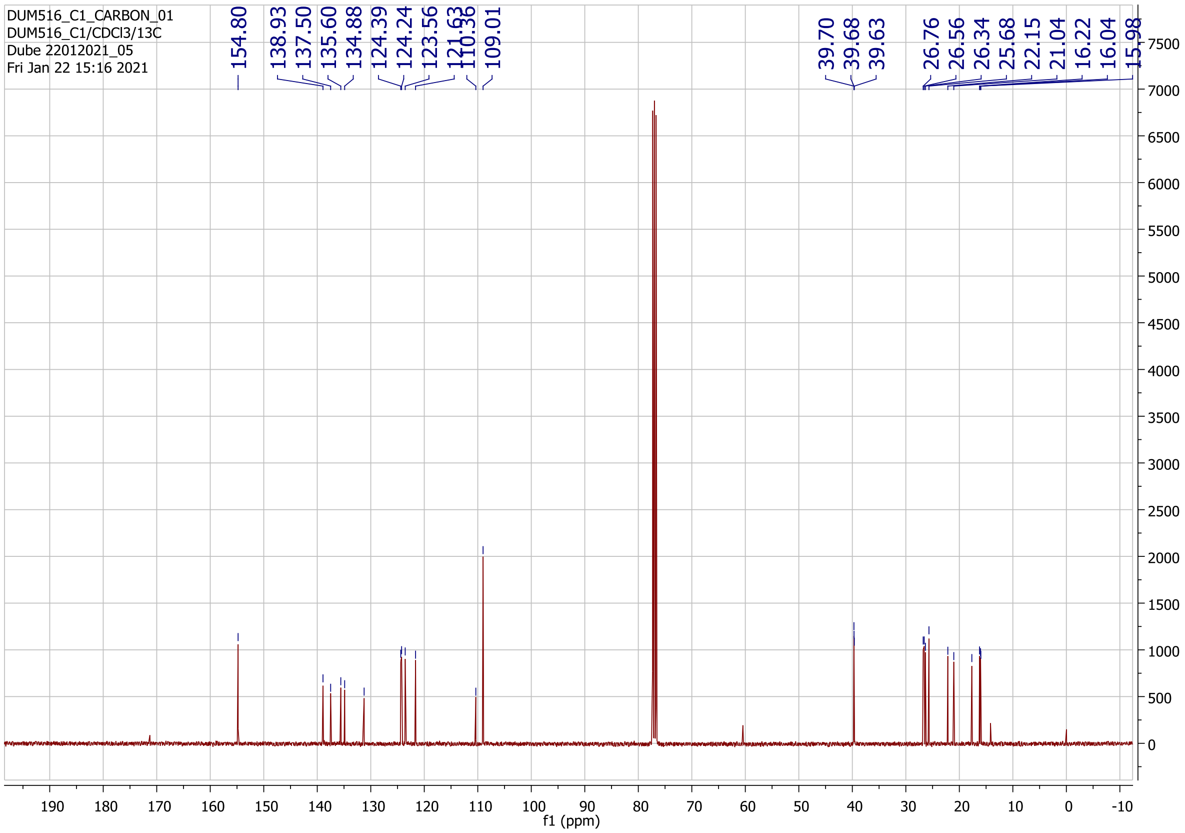

Figure S28: ^{13}C NMR spectrum of geranylgeranyl-2-orcinol (**6**) in $CDCl_3$ (400 MHz, δ in ppm).

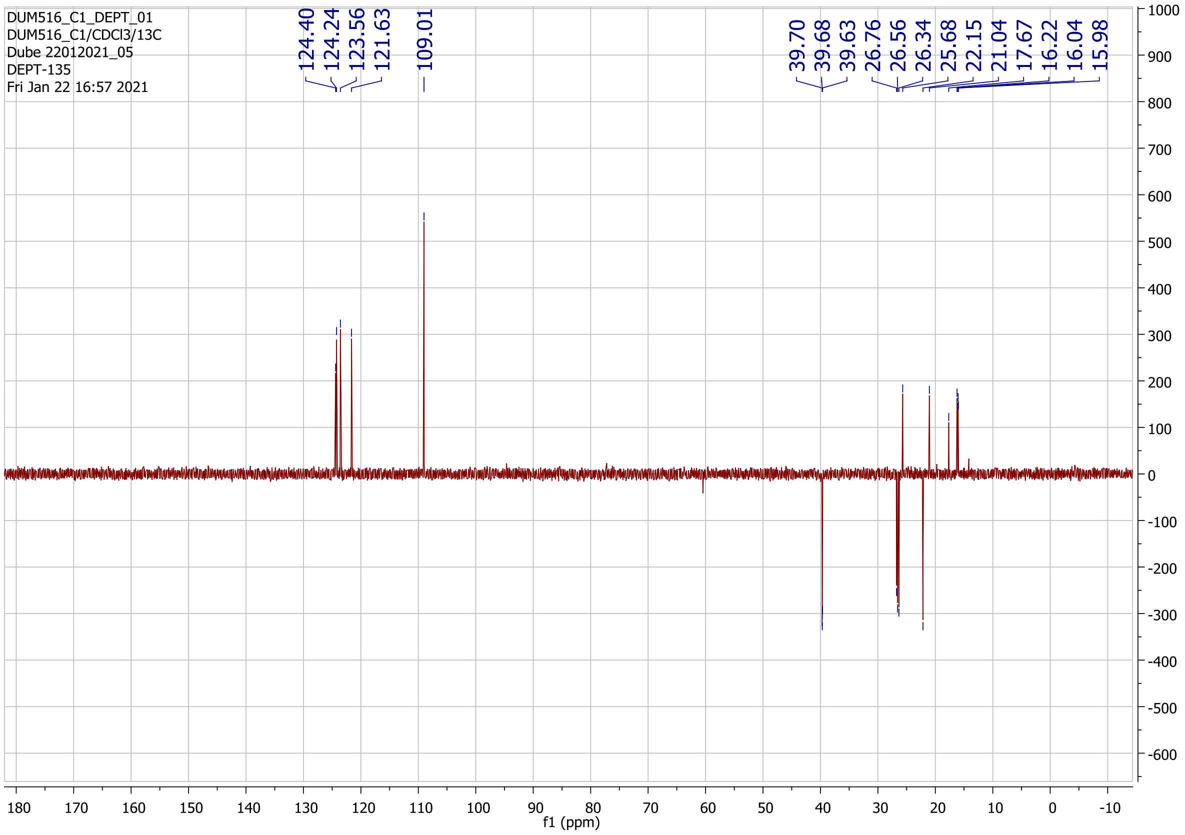

Figure S29: DEPT 135 spectrum of geranylgeranyl-2-orcinol (**6**) in $CDCl_3$ (400 MHz, δ in ppm).

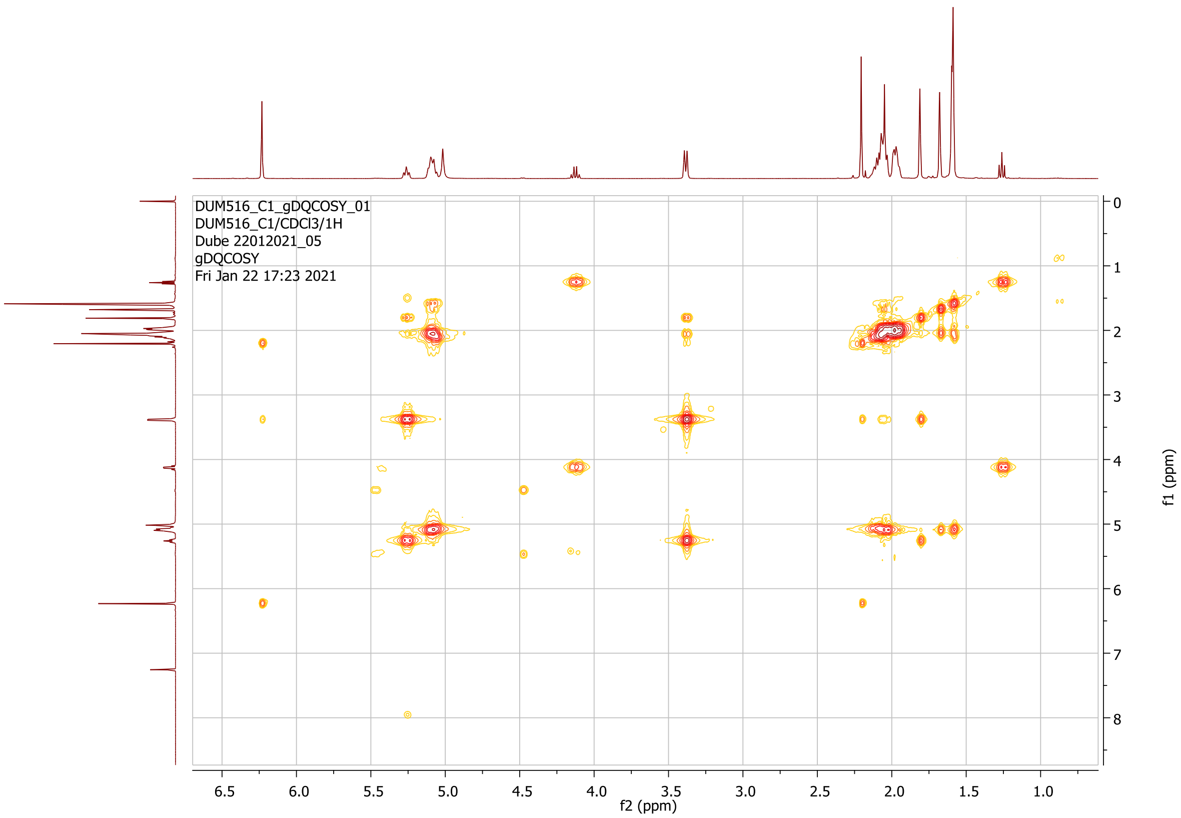

Figure S30: gDQCOSY spectrum of geranylgeranyl-2-orcinol (**6**) in $CDCl_3$ (400 MHz, δ in ppm).

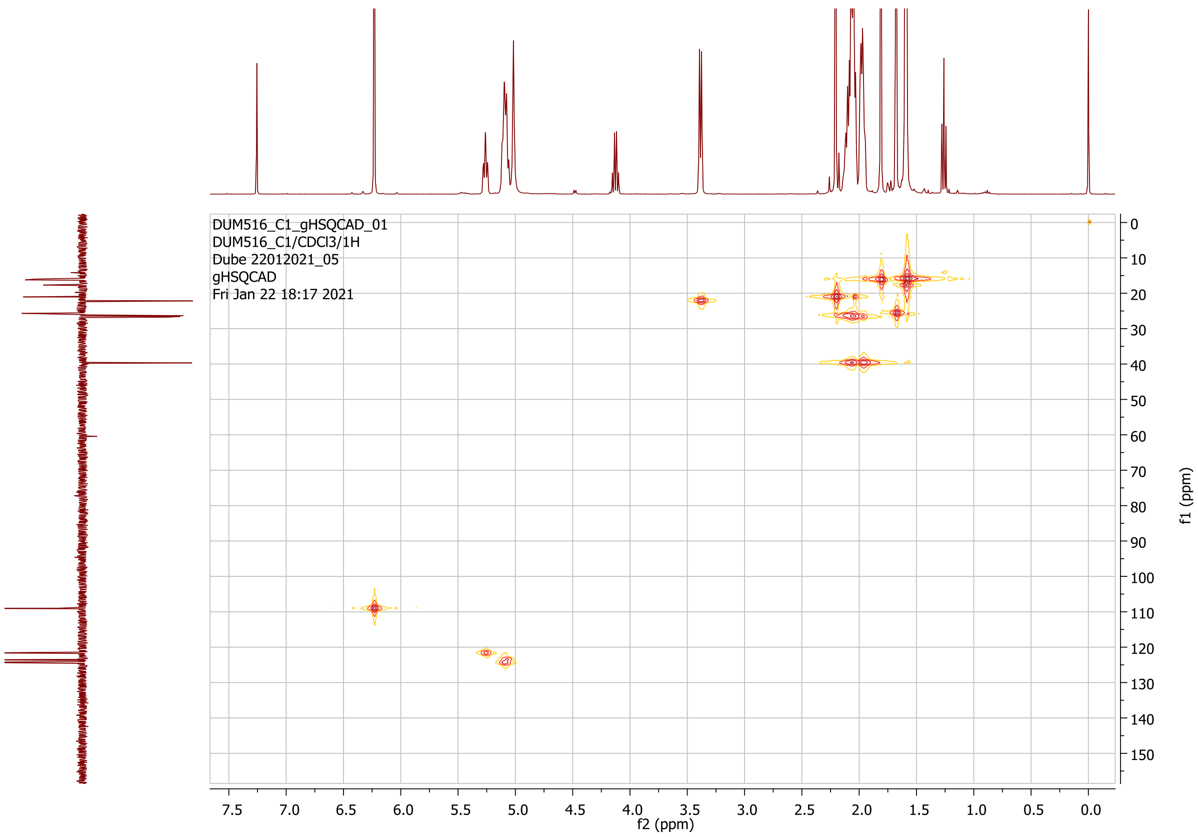

Figure S31: gHSQC spectrum of geranylgeranyl-2-orcinol (**6**) in $CDCl_3$ (400 MHz, δ in ppm).

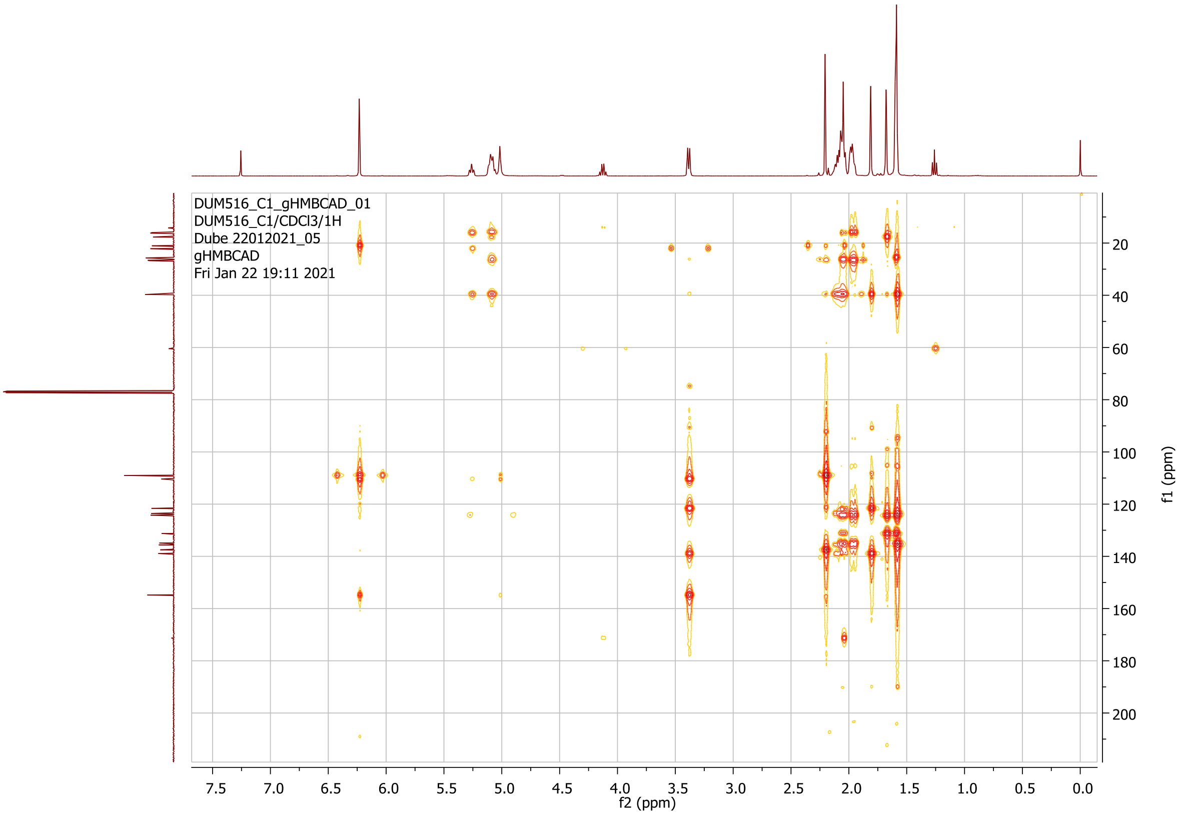

Figure S32: gHMBC spectrum of geranylgeranyl-2-orcinol (**6**) in $CDCl_3$ (400 MHz, δ in ppm).

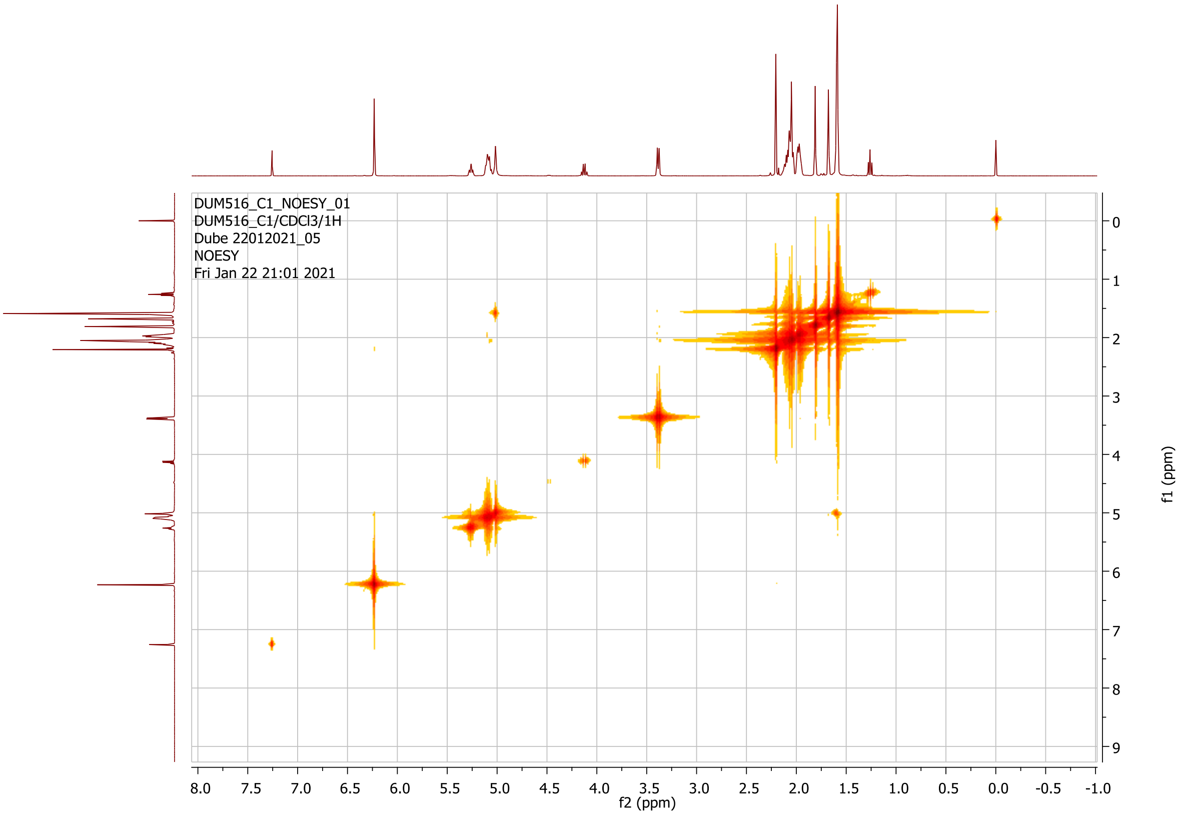

Figure S33: NOESY spectrum of geranylgeranyl-2-orcinol (**6**) in $CDCl_3$ (400 MHz, δ in ppm).

Table S1. ^{1}H (400 MHz, δ in ppm) and ^{13}C (100 MHz, δ in ppm) NMR data of grifolin (**1**) in $CDCl_3$.

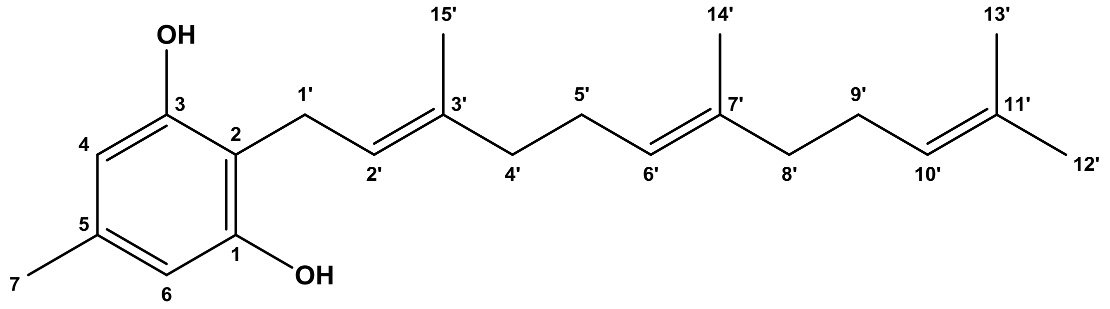

Position	δH (m)	δC (m)
**1**	-	154.8 (s)
**2**	-	110.3 (s)
**3**	-	154.8 (s)
**4**	6.23 (1H, s)	109.0 (d)
**5**	-	137.5 (s)
**6**	6.23 (1H, s))	109.0 (d)
**7**	2.21 (3H,s)	21.0 (q)
OH-**1**[a]<br>OH-**3**[a]	4.97 (2H, s)	-
**1′**	3.39 (2H, d, 7.1)	22.2 (t)
**2′**	5.26 (1H, t, 7.1)	121.6 (d)
**3′**	-	138.9 (s)
**4′**	2.08 – 2.13 (2H, m)	39.7 (t)
**5′**	2.01 – 2.08 (2H, m)	26.7 (t)
**6′**	5.08 (1H, m)	123.6 (d)
**7′**	-	135.6 (s)
**8′**	2.01 – 2.08 (2H, m)	39.6 (t)
**9′**	1.93-2.00 (2H, m)	26.3 (t)
**10′**	5.08 (1H, m)	124.4 (d)
**11′**	-	131.3 (s)
**12′**	1.67 (3H, s)	25.7 (q)
**13′**	1.60 (3H, s)-	17.7 (q)
**14′**	1.58 (3H, s)	16.2 (q)
**15′**	1.81 (3H, s)	16.0 (q)

[a] Signals may be interchanged.

Table S2. ^{1}H (400 MHz, δ in ppm) and ^{13}C (100 MHz, δ in ppm) NMR data of compound **2** in $CDCl_3$.

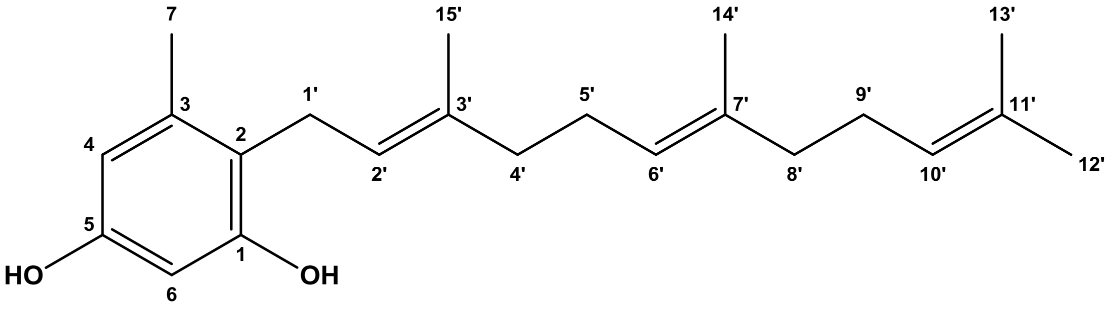

Position	δH (m)	δC (m)
**1**	-	155.4 (s)
**2**	-	117.9 (s)
**3**	-	138.4 (s)
**4**	6.26 (1H, d, 2.4)	109.6 (d)
**5**	-	154.2 (s)
**6**	6.21 (1H, d, 2.5)	101.0 (d)
**7**	2.23 (3H,s)	21.0 (q)
OH-**1**	5.14 (1H,s)	-
OH-**5**	4.61 (1H, s)	-
**1′**	3.29 (2H, d, 6.8)	25.1 (t)
**2′**	5.15 (1H, t, 7.1)	122.0 (d)
**3′**	-	137.7 (s)
**4′**	2.09 (2H, t, 7.5)	39.6 (t)
**5′**	2.01 – 2.06 (2H, m)	26.7 (t)
**6′**	5.07 (1H, m)	123.7 (d)
**7′**	-	135.4 (s)
**8′**	2.01 – 2.06 (2H, m)	39.6 (t)
**9′**	1.93-1.98 (2H, m)	26.4 (t)
**10′**	5.07 (1H, m)	124.4 (d)
**11′**	-	131.3 (s)
**12′**	1.67 (3H, s)	25.7 (q)
**15′**	1.79 (3H, s)	16.0 (q)
**14′**	1.58 (3H, s)	16.2 (q)
**13′**	1.59 (3H, s)-	17.7 (q)

Table S3. ^{1}H (400 MHz, δ in ppm) and ^{13}C (100 MHz, δ in ppm) NMR data of prenyl-2-orcinol (**4)** in $CDCl_3$.

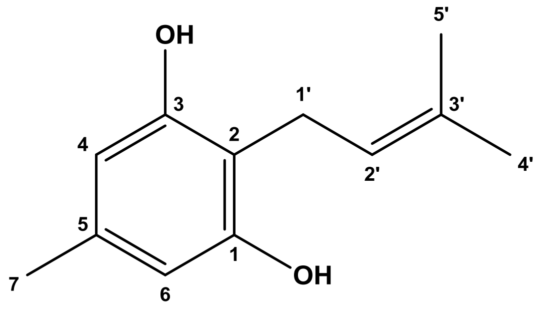

Position	δ_H (m)	δ_C (m)
**1**	-	154.7 (s)
**2**	-	110.4 (s)
**3**	-	154.7 (s)
**4**	6.23 (1H, s)	109.0 (d)
**5**		137.5 (s)
**6**	6.23 (1H, s))	109.0 (d)
**7**	2.20 (3H,s)	21.0 (q)
OH-**1**[a]<br>OH-**3**[a]	5.14 (2H, s)	-
**1′**	3.37 (2H, d, 7.1)	22.2 (t)
**2′**	5.25 (1H, t, 7.1)	121.8 (d)
**3′**	-	135.0 (s)
**4′**	1.74 (3H, s)	25.7 (q)
**5′**	1.81 (3H, s)	17.8 (q)

[a] Signals may be interchanged.

Table S4. ^1H (400 MHz, δ in ppm) and ^{13}C (100 MHz, δ in ppm) NMR data of geranyl-2-orcinol (**5)** in $CDCl_3$.

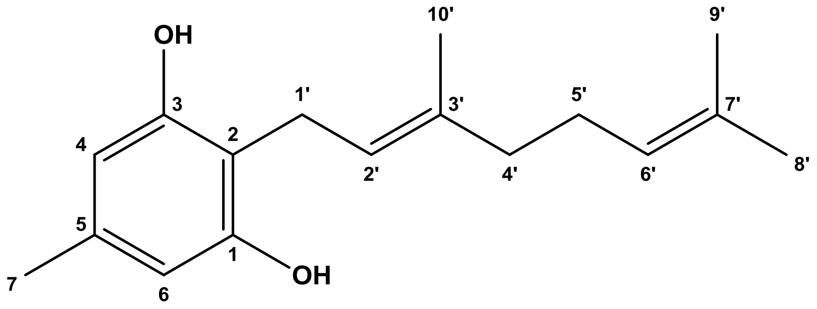

Position	δH (m)*H*	δC (m)
**1**	-	155.0 (s)
**2**	-	110.6 (s)
**3**	-	155.0 (s)
**4**	6.23 (1H, s)	108.8 (d)
**5**		137.4 (s)
**6**	6.23 (1H, s))	108.8 (d)
**7**	2.21 (3H,s)	21.0 (q)
OH-**1**[a]<br>OH-**3**[a]	5.66 (2H, s)	-
**1′**	3.38 (2H, d, 7.1)	22.1 (t)
**2′**	5.26 (1H, t, 7.6)	121.9 (d)
**3′**	-	138.5 (s)
**4′**	2.07 (2H, m)	39.7 (t)
**5′**	2.07 (2H, m)	26.4 (t)
**6′**	5.06 (1H, t, 6.0)	123.8 (d)
**7′**	-	131.9 (s)
**8′**	1.67 (3H, s)	25.6 (q)
**9′**	1.59 (3H, s)	17.7 (q)
**10′**	1.80 (3H, s)	16.1 (q)

[a] Signals may be interchanged.

Table S5. ^{1}H (400 MHz, δ in ppm) and ^{13}C (100 MHz, δ in ppm) NMR data of geranylgeranyl-2-orcinol (**6**) in $CDCl_3$.

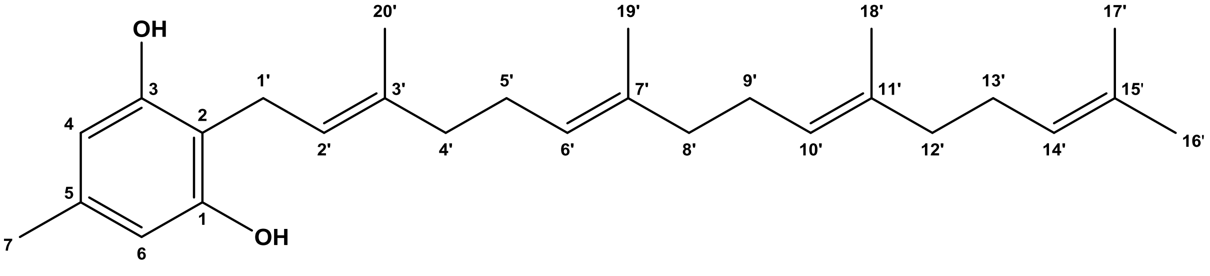

Position	δ_H (m)$_H$	δ_C (m)
**1**	-	154.8 (s)
**2**	-	110.4 (s)
**3**	-	154.8 (s)
**4**	6.23 (1H, s)	109.0 (d)
**5**	-	137.5 (s)
**6**	6.23 (1H, s))	109.0 (d)
**7**	2.21 (3H,s)	21.0 (q)
OH-**1**[a]<br>OH-**3**[a]	5.02 (2H, s)	-
**1′**	3.38 (2H, d, 7.1)	22.2 (t)
**2′**	5.26 (1H, dd, 7.1; 6.4)	121.6 (d)
**3′**	-	138.9 (s)
**4′**	1.98-2.07 (2H, m)	39.7 (t)
**5′**	1.98-2.07 (2H, m)	26.8 (t)
**6′**	5.09 (1H, m)	124.4 (d)
**7′**	-	135.6 (s)
**8′**	1.98-2.07 (2H, m)	39.7 (t)
**9′**	1.98-2.07 (2H, m)	26.6 (t)
**10′**	5.09 (1H, m)	123.6 (d)
**11′**	-	134.8 (s)
**12′**	1.98-2.07 (2H, m)	39.6 (t)
**13′**	1.98-2.07 (2H, m)	26.3 (t)
**14′**	5.09 (1H, m)	124.2 (d)
**15′**	-	131.2 (s)
**16′**	1.68 (3H, s)	25.7 (q)
**17′**	1.59 (3H, s)	17.7 (q)
**18**	1.59 (3H, s)	16.0 (q)
**19′**	1.59 (3H, s)	16.2 (q)
**20′**	1.81 (3H, s)	16.0 (q)

[a] Signals may be interchanged.

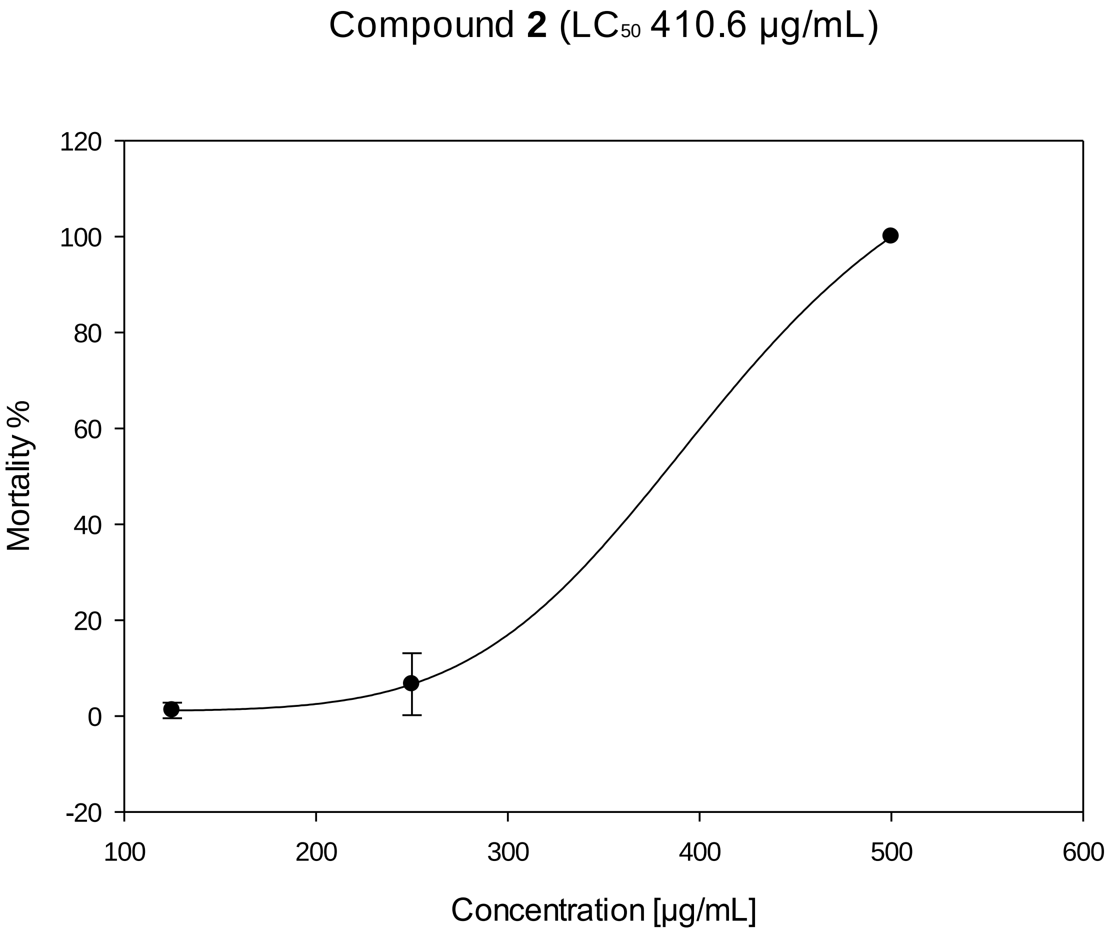

Figure S34: LC_{50} curve for in vitro anthelmintic activity of Compound **2** against *C. elegans*.
